# Prenatal influenza vaccination rescues impairments of social behavior and lamination in a mouse model of autism

**DOI:** 10.1186/s12974-018-1252-z

**Published:** 2018-08-13

**Authors:** Yingying Wu, Fangfang Qi, Dan Song, Zitian He, Zejie Zuo, Yunjie Yang, Qiongliang Liu, Saisai Hu, Xiao Wang, Xiaona Zheng, Junhua Yang, Qunfang Yuan, Juntao Zou, Kaihua Guo, Zhibin Yao

**Affiliations:** 1 Department of Anatomy and Neurobiology, Zhongshan School of Medicine, Sun Yat-sen University, #74, Zhongshan No. 2 Road, Guangzhou, 510080 China; 2 Department of Thoracic Surgery, The First Affiliated Hospital, Sun Yat-sen University, Guangzhou, 510080 China; 3 Guangdong Province Key Laboratory of Brain Function and Disease, Zhongshan School of Medicine, Sun Yat-sen University, #74, Zhongshan No. 2 Road, Guangzhou, 510080 China

**Keywords:** Influenza vaccine, Autism, Cortical layers, Neuronal differentiation, Ikzf1

## Abstract

**Background:**

Prenatal infection is a substantial risk factor for neurodevelopmental disorders such as autism in offspring. We have previously reported that influenza vaccination (VAC) during early pregnancy contributes to neurogenesis and behavioral function in offspring.

**Results:**

Here, we probe the efficacy of VAC pretreatment on autism-like behaviors in a lipopolysaccharide (LPS)-induced maternal immune activation (MIA) mouse model. We show that VAC improves abnormal fetal brain cytoarchitecture and lamination, an effect associated with promotion of intermediate progenitor cell differentiation in MIA fetal brain. These beneficial effects are sufficient to prevent social deficits in adult MIA offspring. Furthermore, whole-genome analysis suggests a strong interaction between Ikzf1 (IKAROS family zinc-finger 1) and neuronal differentiation. Intriguingly, VAC rescues excessive microglial Ikzf1 expression and attenuates microglial inflammatory responses in the MIA fetal brain.

**Conclusions:**

Our study implies that a preprocessed influenza vaccination prevents maternal bacterial infection from causing neocortical lamination impairments and autism-related behaviors in offspring.

**Electronic supplementary material:**

The online version of this article (10.1186/s12974-018-1252-z) contains supplementary material, which is available to authorized users.

## Background

Autism spectrum disorder (ASD) is a psychiatric disorder characterized by severe and pervasive impairments in communication and social interaction and by stereotyped or repetitive behaviors [1, 2]. Studies have revealed that maternal immune activation (MIA) increases the risk for ASD [1, 3]. Currently, the most widely used animal model for MIA is gestational exposure to bacterial or viral infections [4–6]. The literature reveals that a single injection of lipopolysaccharide (LPS) can result in deficits in social interaction, novel object recognition, anxiety-like behavior, and activation of microglia in the fetal brain [5, 7, 8].

Data have confirmed that pregnant women are vulnerable to influenza virus infection [9, 10]. Therefore, influenza vaccination (VAC) is recommended in pregnant women for its efficacy and safety [11]. Our previous findings suggest that prenatal VAC contributes to neurogenesis both in pregnant mice and in their offspring [12–14]. Notably, maternal VAC promotes exploratory behaviors in offspring and blocks subsequent LPS challenge from causing spatial cognitive impairments in Morris water maze performance [12]. These effects are also accompanied by a decrease in proinflammatory cytokines, reminiscent of the inflammatory response induced by LPS treatment [3, 5]. Mechanistically, VAC promoted M2 microglial/macrophage polarization via neuronal secretion of BDNF and microglia expressing IGF-1, thereby developing a pro-neurogenic niche [13, 15]. These results revealed that whether VAC could prevent LPS-induced autism-related behaviors and defects in neurodevelopment is worthy of exploration. Therefore, we assume that VAC pretreatment in early pregnancy has the potential to exert protective effects against autism-like behaviors and on the developing brain in an LPS-induced MIA model.

Previous neuroimaging studies of autistic patients and animal models have revealed several anatomical alterations that may be related to their behavioral abnormalities. These changes include altered total brain volume and cortical plate (CP) thickness, a thinner corpus callosum, and malformations in cortex lamination [16–18]. We then explored whether VAC protected the thickness of neocortex and lamination in the fetal brain of an MIA-related model. Furthermore, it is well known that radial glial cells (RGCs) and intermediate progenitor cells (IPCs) are primary cortical precursor cells that are located in the VZ/SVZ and differentiate into mature neurons [19, 20]. Notably, precursor cell proliferation and differentiation are most intense during a crucial period of time before birth. During that period, RGCs and IPCs form substantial numbers of neurons in an inside-out pattern that gives rise to neocortical lamination, suggesting a tight connection between neuronal differentiation and lamination [21, 22].

In this study, we explored the influence of VAC on autism-like behaviors and on the laminar organization of cortical neurons in an LPS-induced MIA mouse model. As hypothesized, we found that VAC improved social deficiency in adult MIA offspring. Further, VAC prevented the LPS-induced aberrations in brain morphology and laminar cytoarchitecture, probably by counteracting abnormal neuronal differentiation. Finally, RNA-Seq was carried out to explain the biological mechanism at the genome level, and Ikzf1 (IKAROS family zinc-finger 1) was proved to be tightly correlated with autism-like behavior through its regulation of neuronal differentiation. Meantime, VAC also attenuated the central inflammatory response to LPS challenge. Collectively, these data suggest that VAC is a potential precaution against maternal bacterial infection.

## Methods

### Animals

**Fig. 1:**
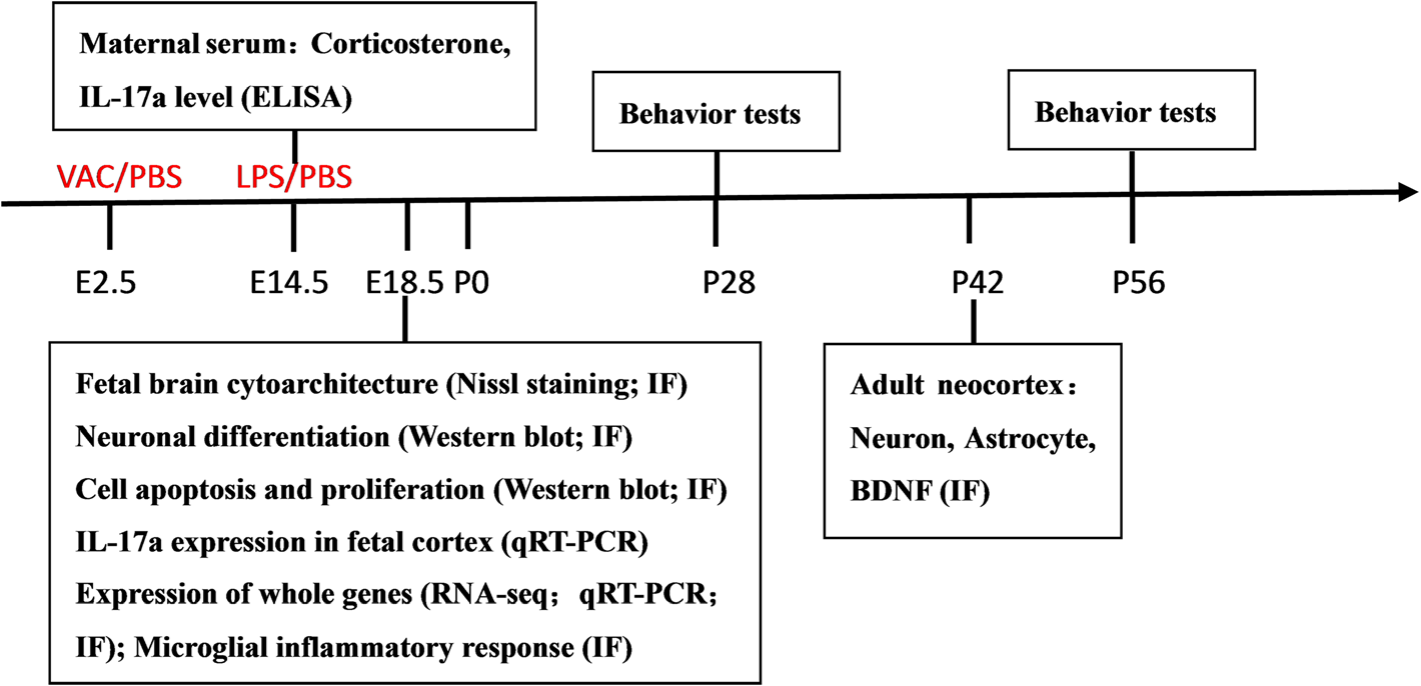
Experimental flowchart. Pregnant C57BL/6 mice were treated with influenza vaccine (VAC) or sterilized PBS at E2.5 and with sterilized LPS or PBS at E14.5. Two hours after the LPS or PBS injection at E14.5, maternal serum corticosterone and IL-17a levels were detected. The fetal brains were removed for morphology, protein, and gene detection at E18.5. The offspring were submitted to behavior tests at 4 weeks (P28) and 8 weeks (P56) after birth, and the brains were removed for immunofluorescence staining at 6 weeks (P42)

C57BL/6 mice were purchased from the Laboratory Animal Center of Sun Yat-sen University (Guangzhou, China). Mice harboring green fluorescent protein (GFP) under control of the Cx3cr1 promoter (B6.129P-Cx3cr1tm1Litt/J) were purchased from The Jackson Laboratory (stock no. 005582). To generate timed pregnancies, we housed pairs of females with single males overnight. The mice were separated at noon the next day. The time when a vaginal plug was observed was defined as embryonic day 0.5 (E0.5), and the delivery day was designated postnatal day 0 (P0). Pups were weaned at postnatal day 21 and caged separately in groups of four to five littermates by sex. To rule out maternal variables, all experiments used one pup per dam. The basic experimental operation flow is shown in Fig. 1. All animals were housed under standard illumination parameters (12-h light/dark cycle) and received water and food ad libitum. All animal experiments were approved by the Institutional Animal Care and Use Committee of Sun Yat-sen University.

### Influenza vaccination

Mice were randomly treated with split inactivated influenza vaccine (VAC) or sterilized PBS (PBS) by intramuscular (i.m.) injection in their quadriceps at a single dose of 3 μg/mouse during the first trimester (E2.5). The inactivated influenza vaccines (split virion) contain the following: an A/California/7/2009 (H1N1) pdm09-like virus, an A/Hong Kong/4801/2014 (H3N2)-like virus, and a B/Brisbane/60/2008-like virus (B/Victoria lineage). Excipients in vaccines include NaH_2_PO_4_, NaH_2_PO_4_, and NaCl. Vaccines produced by Changchun Institute of Biological products were obtained from the Centers for Disease Control and Prevention (CDC, Guangdong, China) and had previously been shown to be immunogenic and safe.

### Maternal immune activation

**Fig. 2:**
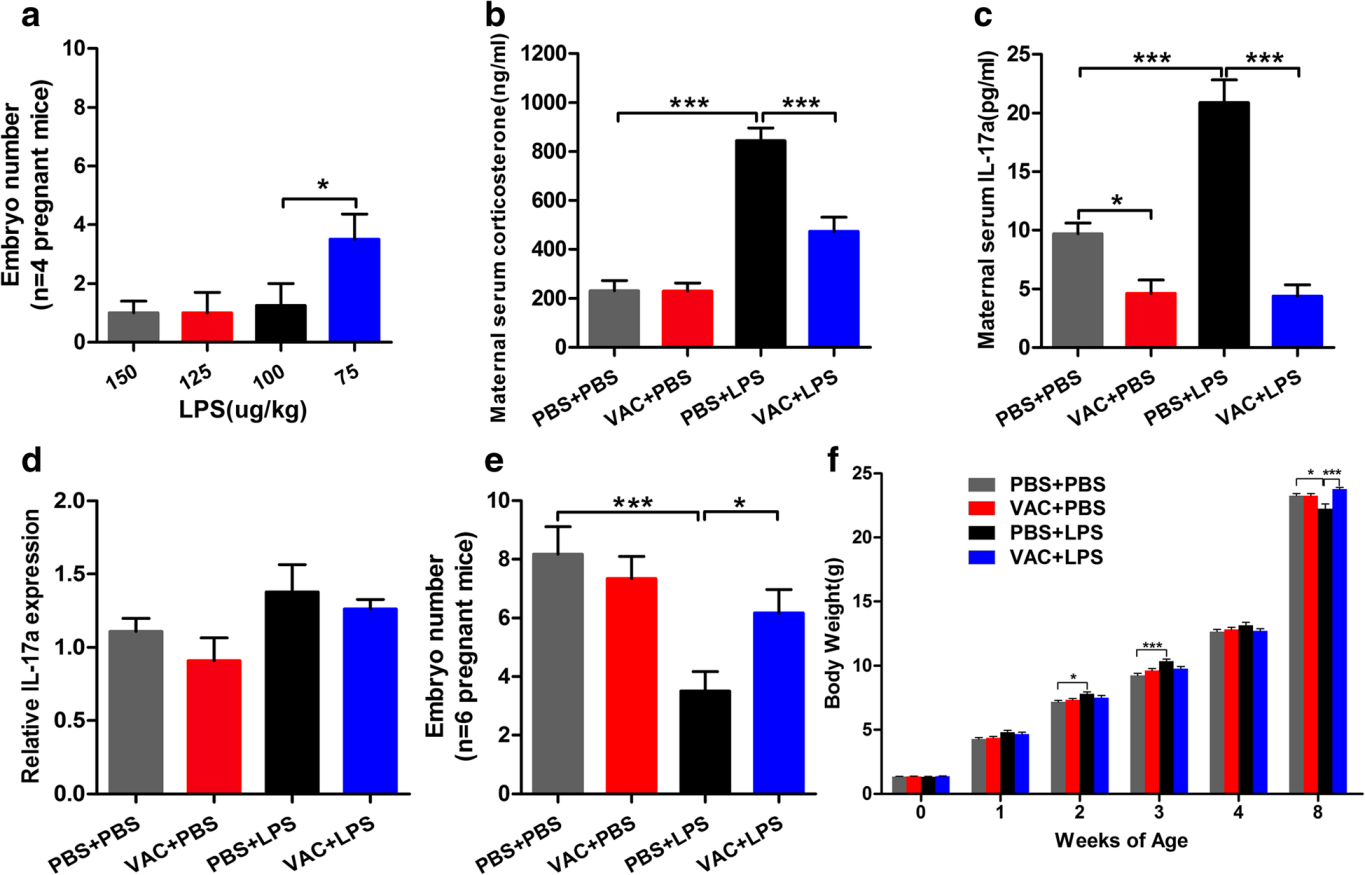
Effects of maternal VAC and MIA on maternal inflammatory response, litter characteristics, and body weight. **a** Pregnant mice were treated with LPS at doses of 150, 125, 100, and 75 μg/kg LPS, and viable embryos were obtained at the dose of 75 μg/kg (*F*_3,12_ = 2.979, *p* < 0.05). *n* = 4 mice/group (one-way ANOVA and Bonferroni post hoc test). **b** A two-way ANOVA for corticosterone level revealed a significant effect of MIA (*F*_1,19_ = 76.971, *p* < 0.001) and VAC (*F*_1,19_ = 14.568, *p* = 0.001) and interaction of MIA × VAC (*F*_1,19_ = 14.369, *p* = 0.001). Subsequent analyses revealed that the levels of corticosterone were increased in the maternal serum at 2 h after LPS injection (*p* < 0.001) and that VAC pretreatment prevented this effect (*p* < 0.001). *n* = 5–7 mice/group. **c** A two-way ANOVA for IL-17a level showed a significant effect of MIA (*F*_1,21_ = 15.806, *p* = 0.001) and VAC (*F*_1,21_ = 60.667, *p* < 0.001) and a significant interaction of MIA × VAC (*F*_1,21_ = 15.806, *p* = 0.0004). A post hoc test showed that LPS increased the level of IL-17a in the maternal serum at E14.5 compared to controls (*p* < 0.001), and VAC pretreatment prevented this effect (*p* = 0.024), but there were no significant effects on IL-17a expression in the fetal cortex at E18.5 (*p* > 0.05) (**d**); *n* = 5–7 mice/group. **e** A two-way ANOVA for the number of embryos revealed a significant effect of MIA (*F*_1,20_ = 13.344, *p* = 0.002) and a significant interaction of MIA × VAC (*F*_1,20_ = 4.804, *p* = 0.04). A post hoc test showed that MIA significantly reduced the average number of embryos (*p* = 0.01) and VAC pretreatment rescued the fetal loss (*p* = 0.028). *n* = 6 mice /group. **f** Offspring were weighed at the indicated times. *n* = 6 mice/group (repeated-measures ANOVA). *p < 0.05, ***p < 0.001; the results are all shown as the mean + s.e.m.

The potassium salt of LPS (*Escherichia coli* O111:B4; L2630; Sigma-Aldrich) was used to induce MIA, but the recommended dose was not determined a priori, as previous reports have used doses from 120 to 300 μg/kg [8, 23]. We injected pregnant mice with a range of LPS concentrations at E14.5 and found that 150, 125, and 100 μg/kg induced abortion or resorption of the fetuses, but viable offspring were obtained with 75 μg/kg LPS (Fig. 2a; *F*_3,12_ = 2.979, *p* < 0.05). Maternal serum corticosterone was evaluated at 2 h after injection of 75 μg/kg LPS, and the level of corticosterone was increased after LPS exposure, suggesting that the hypothalamic-pituitary-adrenal (HPA) axis was activated by a dose of 75 μg/kg LPS (Fig. 2b).

Thus, 75 μg/kg LPS or saline was administered to pregnant dams by intraperitoneal (i.p.) injection at E14.5. This time point is in the second half of mouse gestation, which roughly corresponds to the late first or early second trimester in human gestation, when infections confer the greatest risk of ASD in human offspring [7, 24]. At this stage, the pregnant dams were divided into four treatment groups: PBS+PBS, VAC+PBS, PBS+LPS, and VAC+LPS. The number of pups for each pregnant mouse was recorded on P0, and the weight changes from 0 to 8 w of age in each group were also recorded.

### Enzyme-linked immunosorbent assay

Two hours after LPS injection, serum was separated from trunk blood by centrifugation at 2500 rpm for 15 min and was subsequently stored at − 80 °C until use. Serum corticosterone levels were measured using a corticosterone ELISA kit (Abcam, Cambridge, UK) according to the manufacturer’s instructions. The concentration of serum IL-17a was measured using a Mouse SimpleStep ELISA kit (Abcam).

### Behavioral tests

One limitation of the current MIA models is that they focus on ASD-related phenotypes once the offspring reach early adulthood, whereas overt ASD symptoms typically emerge during childhood. Thus, we chose two time points, 4 and 8 w, for testing ASD behavior. Behavioral testing of the offspring was performed during the light period (between 10:00 and 16:00 h). Before the assay began, the mice were moved to a holding room in the behavioral testing area, where they were kept for at least 1 h. The apparatuses were cleaned with 70% ethanol and allowed to dry completely before each test.

### Open field test (OFT)

The animals were individually placed in the center of the open field arena (50 × 50 × 50 cm) with the illumination located on the top surface of the equipment. The spontaneous locomotor activity of each animal was recorded for 10 min with the TopScan™ 2.0 system (Clever Sys. Inc.). Total distance, distance traveled in the peripheral and center areas, time spent in the center area, and number of entries into the center area were recorded automatically.

### Elevated plus maze

Each animal was placed in the center of the elevated plus maze (EPM), with its head facing one of the open arms, and was allowed to freely explore the apparatus for 5 min. Spontaneous locomotor activity was recorded for 10 min with the TopScan™ 2.0 system (Clever Sys. Inc.). The time spent in and the numbers of entries into the open arms and closed arms were analyzed.

### Three-chamber social approach and social novelty tests

Mice were tested as previously described [25]. Animals were placed in the center of a clear acrylic arena (59 × 39 × 22 cm) that was divided into three equal compartments. Testing consisted of three phases: 5 min of acclimation to the empty arena, 10 min of sociability testing, and 10 min of social novelty testing. Each side compartment contained a clear plexiglass cylinder (7.5 cm in diameter, with several holes to allow nose contact). At the beginning of the test, each animal was placed in the central compartment and allowed to explore for 5 min (habituation). Then, an unfamiliar young (4 weeks) C57BL/6 mouse was placed in one of the cylinders for sociability testing. Finally, another C57BL/6 mouse of the same age was placed in the other cylinder for social novelty testing. Time spent in each chamber was recorded with SuperMaze animal behavior video analysis software (Shanghai Xinruan Information Technology Co., Ltd., China).

### Tail suspension test

The tail suspension test (TST) was performed as previously described [26]. Animals were suspended in the air using adhesive tape wrapped around the tail and fixed to a wire 25 cm above a wooden surface. The immobility time in a 5-min period was measured with SuperTst High-throughput Tail Suspension Test Analysis (Shanghai Xinruan Information Technology Co. Ltd., China).

### Forced swim test

The forced swim test (FST) was performed as previously reported [26]. Mice were gently placed in a glass beaker (diameter, 15 cm; height, 25 cm), filled with 14 cm of water at room temperature (25 °C). Immobility in a 5-min period was recorded with SuperFst High-throughput Forced Swim Test software (Shanghai Xinruan Information Technology Co. Ltd., China). At the end of the test, the animals were dried with a paper towel and placed in a holding cage with normal bedding.

### Western blot

For Western blotting, mice were sacrificed under deep anesthesia at E18.5. The cortex was immediately removed from the brain of each embryo and processed into tissue homogenate in ice-cold RIPA buffer (Beyotime Biotechnology, Wuhan, China) containing phenylmethylsulfonyl fluoride (PMSF) using a homogenizer. After centrifugation (4 °C, 12000 rpm, 15 min), the supernatant was subdivided and stored at − 80 °C for further measurements. A bicinchoninic acid (BCA) protein assay (Beyotime Biotechnology, Wuhan, China) was used to measure the protein concentrations of the samples. The supernatant was mixed with loading buffer and boiled for 5 min. Equivalent amounts of protein extracts were separated on 15% or 10% SDS-polyacrylamide gels and electrophoretically transferred to polyvinylidene fluoride (PVDF) membranes. The membranes were blocked with 5% BSA for 1 h and then incubated overnight at 4 °C with the primary antibodies. The following primary antibodies were used at the indicated concentrations: anti-cleaved caspase-3 (1:1000, Cell Signaling Technology), anti-Pax-6 (1:1000, Abcam), and anti-β-actin (1:1000, Cell Signaling Technology). HRP-conjugated secondary goat anti-rabbit or goat anti-mouse antibodies (Sigma-Aldrich, St. Louis, MO, USA) were used at 1:10000. Protein bands were visualized using enhanced chemiluminescence (Amersham Biosciences). The protein expression levels of cleaved caspase-3, Pax-6, and β-actin were semiquantitatively evaluated by using ImageJ software.

### Tissue preparation

Pregnant dams were deeply anesthetized at E18.5, and uteri were removed and placed on ice. Then, the fetuses were transcardially perfused with heparinized saline followed by cold 4% paraformaldehyde (PFA) in 0.1 M phosphate buffer (pH = 7.2). The fetal brains were removed, fixed in 4% PFA overnight, and equilibrated in 10, 20, and 30% sucrose for 24 h each at 4 °C. Then, 40-μm-thick coronal sections for immunofluorescence and 20-μm-thick coronal sections for Nissl staining were obtained on a freezing microtome (Leica SM2000R) and stored in PBS at 4 °C.

For adult neocortex analysis, offspring were deeply anesthetized at 6 weeks and transcardially perfused, and serial coronal sections (40 μm) were collected for immunostaining by the same method used for fetuses.

### Nissl staining

Four to five E18.5 fetal brains from different pregnant mice in each group were collected, and two coronal sections near the site, approximately 1480 μm from the front of the olfactory bulb, were analyzed. Cresyl violet/Nissl staining was performed by standard protocols: 20-μm-thick sections were stained with 0.5% cresyl violet and washed in distilled water, then dehydrated with a graded ethanol series and cleared in xylene.

Images were observed and captured with a BX63 Olympus microscope. The measurements of the dorsal and lateral neocortex were based on the previous report [27]. Total neocortical thickness was measured along a line orthogonal to the superficial pia and lateral ventricular surfaces, as the red line shown in Fig. 5b. Using ImageJ software, neocortical thickness was measured by the “measure” function on straight-line selections extending through the neocortex. Similarly, coronal sections in the same sites were stained for Tbr1 antibody to more precisely define the thickness of the CP. Then, using ImageJ software, CP thickness (layer II–VI) was measured along a line orthogonal to the superficial pia and lateral ventricular surfaces.

### Immunofluorescence (IF)

Coronal sections of brains from mouse pups at E18.5 or 6 weeks after birth, which were randomly selected from at least three different pregnant mice, were stained with various antibodies. Slices were blocked in 1% BSA containing 10% normal goat serum and 0.25% Triton X-100 (Sigma) at 37 °C for 1 h and then incubated with primary antibodies overnight at 4 °C. The next day, the sections were incubated with the secondary antibodies at 37 °C for 2 h. Slices were stained with Hoechst nuclear (1:1000, H3570, Enzo Life Sciences) for 1 min and mounted with Immu-Mount (Thermo).

The primary antibodies included rabbit anti-TBR1 (1:200, ab31940, Abcam), mouse anti-SATB2 (1:200, ab51502, Abcam), rabbit anti-cleaved caspase-3 (1:400, Asp175, Cell Signaling Technology), mouse anti-proliferating cell nuclear antigen (PCNA) (1:100, 60097-1-Ig, Proteintech), rabbit anti-TBR2 (1:1000, ab183991, Abcam), rabbit anti-Iba-1 (1:1000, 019-19741, Wako Chemicals), rat anti-CD68 (1:400, MCA1957, Bio-Rad), mouse anti-NeuN (1:1000, MAB377, Chemicon International), mouse anti-GFAP (1:5000, G3894, sigma), rabbit anti-BDNF (1:400, sc-20,981, Santa Cruz), and rabbit anti-Ikaros (1:100, #14859, Cell Signaling Technology). The following fluorescent secondary antibodies were used: Alexa Fluor 488-conjugated donkey anti-rat, Alexa Fluor 555-conjugated goat anti-rabbit, and Alexa Fluor 488-conjugated goat anti-mouse (1:400; Invitrogen). The negative controls were stained with secondary antibodies alone to assess nonspecific labeling.

Images of stained brain slices were obtained using a confocal microscope (LSM780; Carl Zeiss), with the same parameters for each specific signal. Cells were counted by the optical-fractionator method with a stereology system (Stereo Investigator, MicroBrightField, Williston, USA). We measured the actual section thickness and defined appropriate guard zones at the top and the bottom of the section to avoid oversampling. Measurements were made in an equidistant series of six coronal sections (240 μm apart) with a random starting point, spanning the entire rostrocaudal extent of the cortex and VZ/SVZ.

For BDNF in situ densitometric analysis, sections were studied under × 20 optical fields in the cerebral cortex. Approximately four images were captured per animal. The BDNF signal was measured as an integrated density value using the software ImageJ. Integrated density is the product of area × mean gray value, which measures the signal intensity in a defined area; an equal area with a BDNF-positive signal in each image was selected using the “threshold mode” in ImageJ and subtraction of the background, and data were expressed as arbitrary density units (IntDen).

### Gene expression

Total RNA was extracted from the fetal cortex using TRIzol Reagent (Invitrogen) and processed for RNA-Seq. Remaining total RNA was used to synthesize cDNA with reverse transcriptase M-MLV (Invitrogen) for validation of candidate genes by qRT-PCR. RNA-Seq was performed on a total of 12 mice (three mice per group); detailed run and analysis procedures can be found in the Additional file 1. Quantitative RT-PCR (qRT-PCR) was carried out with the QuantiTect SYBR Green PCR Kit (Qiagen) using the Roche LightCycler® 480 System (Roche, USA) using specific gene primers. The gene expression levels were normalized to those of Gapdh. Quantification cycle (Cq) values obtained by the Lightcycler and delta delta Cq values were calculated to determine relative gene expression values compared with the control.

### Statistical analyses

All the data were processed using SPSS version 22.0 for Windows (SPSS, Inc., Chicago, IL, USA). Data for embryo numbers when applying different doses of LPS were analyzed using one-way analysis of variance (ANOVA) followed by the Bonferroni post hoc test. Body weight data were analyzed using two-way (VAC × LPS) repeated-measures ANOVA followed by the Bonferroni post hoc test. Data from the remaining tests were analyzed using two-way (VAC × LPS) ANOVA followed by the Bonferroni post hoc test. When warranted, planned contrast analyses (Student’s *t* tests) were performed when there were no significant main effects identified via ANOVA. The data are presented as the means + SEMs, and *p* values less than 0.05 were considered to indicate statistically significant differences.

## Results

### VAC counteracted the LPS-induced maternal immune activation and decrease in embryo number

Maternal serum levels of corticosterone and of the pro-inflammatory cytokine IL-17a were evaluated at 2 h after 75 μg/kg LPS injection at E14.5. The data revealed increases in the corticosterone and IL-17a levels of LPS-exposed dams compared to those of the controls at E14.5, and VAC pretreatment alleviated these effects (Fig. 2b, c), suggesting that vaccination at E2.5 could reduce the inflammatory response to LPS at E14.5 in the dams. However, IL-17a expression in the fetal cortex at E18.5 did not differ significantly among the groups (Fig. 2d). MIA is associated with spontaneous pregnancy loss and decreased fetal growth [28]. Our data revealed a significant reduction in the number of embryos after exposure to LPS, whereas VAC attenuated this effect (Fig. 2e). The body weights of the offspring were also measured weekly during postnatal weeks 0–8 (Fig. 2f). Interestingly, LPS caused a dual effect on body weight, i.e., higher weights than the controls at 2 and 3 weeks but lower weights than the controls at 8 weeks. Notably, VAC reversed the LPS-induced effects at 8 weeks (Fig. 2f). These results suggested that VAC prevented abortion of the fetuses and improved the physical condition of offspring subjected to MIA, which may be related to the reduced maternal inflammatory response.

### Pretreatment with VAC improved social behavior in MIA offspring

#### Anxiety-related behavior

**Fig. 3:**
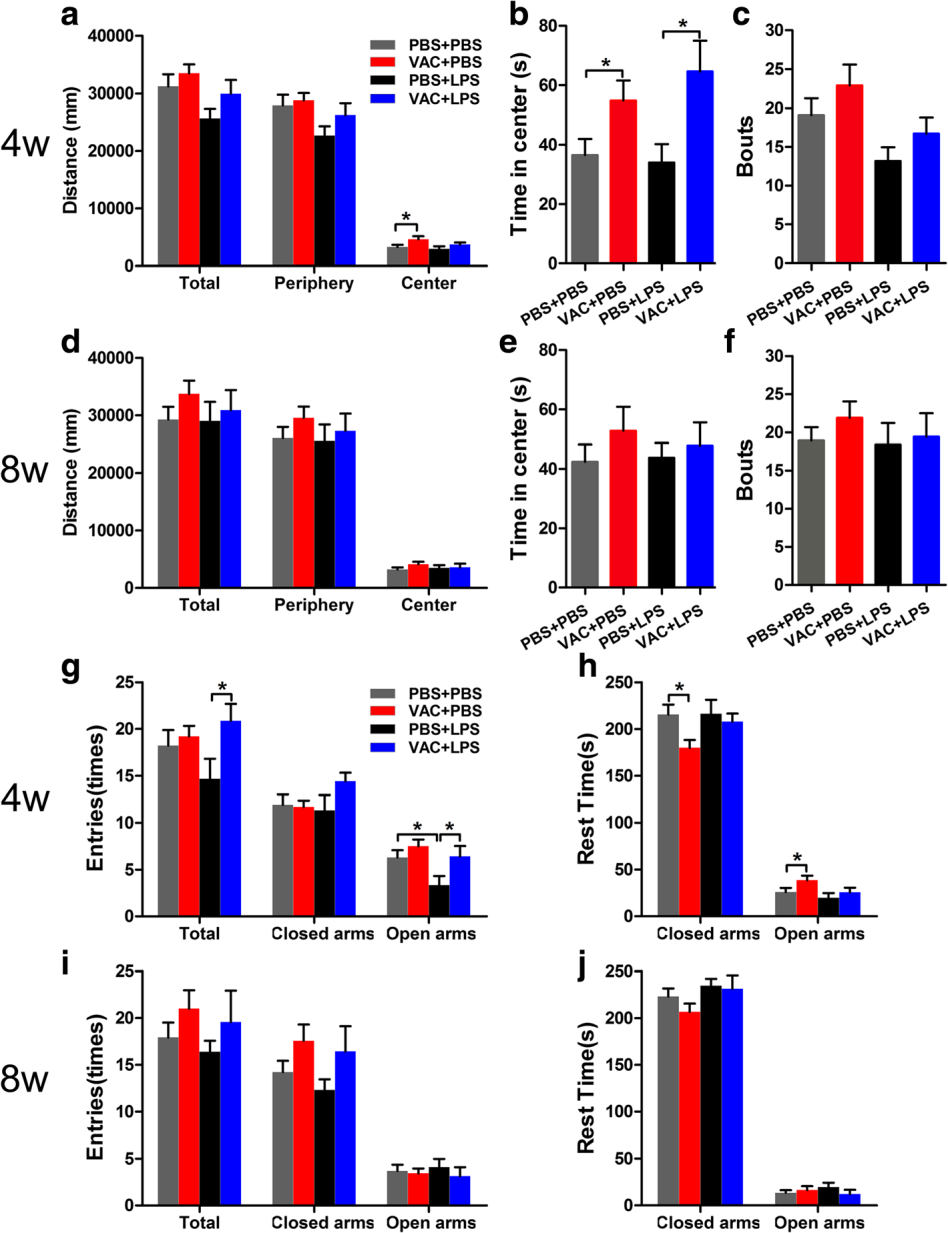
Effects of maternal VAC and MIA on anxiety-related behaviors at 4 and 8 w postnatally. **a–c** In the OFT at 4 w, a two-way ANOVA for distance traveled in the center revealed a significant effect of VAC (*F*_1,32_ = 4.777, *p* = 0.036). Similarly, for time in the center, there was a significant effect of VAC (*F*_1,32_ = 10.988, *p* = 0.002). Subsequent analyses verified that VAC pretreatment increased the distance that mice walked in the center area compared with the control treatment (*p* = 0.026) and increased the time spent in the center (*p* = 0.014). However, there was no significant effect of MIA in the OFT. **g** In the EPM at 4 w, a two-way ANOVA of entries into open arms revealed main effects of MIA (*F*_1,32_ = 4.932, *p* = 0.036) and of VAC (*F*_1,32_ = 5.539, *p* = 0.036). The numbers of entries into the open arms were lower in MIA mice than in controls (*p* = 0.028) but were increased by VAC pretreatment (*p* = 0.041). **h** In the EPM, rest time in the open arms at 4 w was increased by VAC compared to the control treatment (*p* = 0.047). By contrast, there were no significant changes in the open field (**d–f**) or the elevated plus maze (**i** and **j**) at 8 w. *n* = 8–13 mice/group; *p < 0.05 (two-way ANOVA and Bonferroni post hoc test). The results are all shown as the mean + s.e.m.

Given that mood and anxiety disorders have been shown to have high comorbidity with ASD [29], we first measured anxiety-related behaviors with the OFT and EPM at 4 and 8 weeks. The results revealed that MIA decreased the number of entries into the open arms of the EPM and showed a trend of decreasing total distance in center of OFT compared to controls (*p* = 0.082) at 4 w (Fig. 3a, g). Importantly, VAC completely prevented these MIA-induced alterations. Likewise, in line with our previous study [12], at 4 w, VAC mice spent more time in the center of OFT and the open arms of EPM than the corresponding controls did, implying that the influenza vaccine indeed promotes exploratory behavior in adolescent offspring (Fig. 3b, h). Furthermore, we also explored the behavior of 8-week-old mice in the OFT and EPM to supplement our previous study [12]. However, there were no group differences among the 8-week-old mice on the OFT (Fig. 3d–f) or the EPM (Fig. 3i, j), suggesting that the prenatal manipulations produce alterations in locomotor activity and anxiety specifically during the early stages of development.

#### Social interaction behavior

**Fig. 4:**
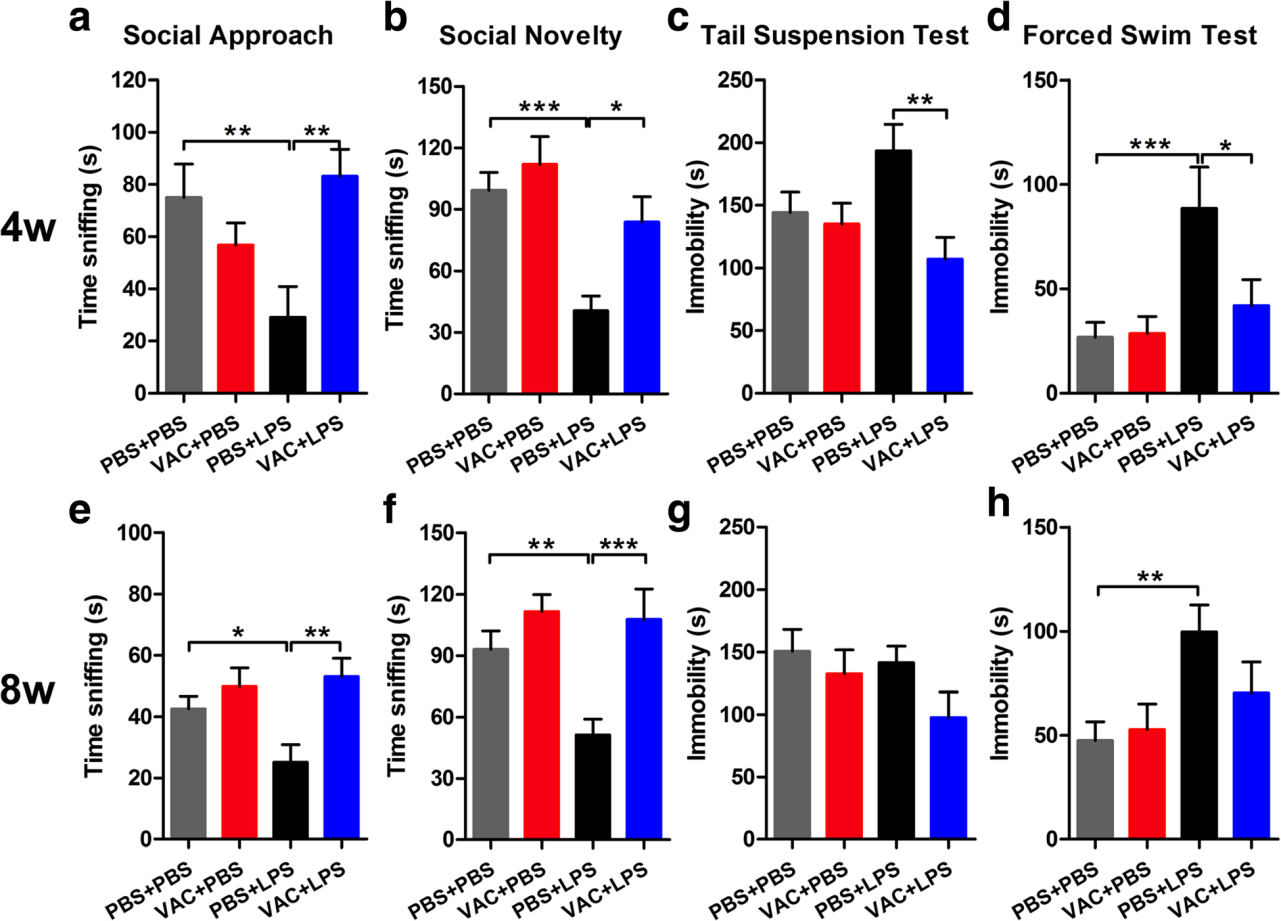
Effects of maternal VAC and MIA on social interaction and depression-related behaviors at 4 and 8 w postnatally. **a**, **e** In social approach, a two-way ANOVA of sniffing time showed a significant interaction of MIA × VAC (*F*_1,33_ = 8.814, *p* = 0.006) at 4 w and a main effect of VAC (*F*_1,36_ = 10.079, *p* = 0.003) at 8 w. **b**, **f** For sniffing time in social novelty, there were main effects of MIA (*F*_1,33_ = 14.394, *p* = 0.001) and VAC (*F*_1,33_ = 5.982, *p* = 0.02) at 4 w and significant effect of MIA (*F*_1,36_ = 5.344, *p* = 0.027) and VAC (*F*_1,36_ = 14.416, *p* = 0.001) at 8 w. Post hoc comparisons revealed that VAC pretreatment could reverse the MIA-induced decreases in time spent sniffing the social stimulus (**a**, **e**) and the novel stimulus (**b**, **f**) by MIA offspring at 4 and 8 w. MIA did not affect the immobility time of the mice in the TST at 4 w (**c**) and 8 w (**g**). **d**, **h** In the FST, a two-way ANOVA of immobility time revealed a significant effect of MIA (*F*_1,32_ = 11.540, *p* = 0.002) and a significant interaction of MIA × VAC (*F*_1,32_ = 4.775, *p* = 0.036) at 4 w, as well as a main effect of MIA (*F*_1,36_ = 8.205, *p* = 0.007) at 8 w. Comparison confirmed that in the FST, MIA mice spent more time immobile than the controls; VAC rescued these effects at 4 w (**d**) but not at 8 w (**h**). *n* = 8–13 mice/group; *p < 0.05, **p < 0.01, ***p < 0.001 (two-way ANOVA and Bonferroni post hoc test). The results are all shown as the mean + s.e.m.

Previous studies demonstrated that MIA offspring have impairments in social interactions, which model the core behavioral deficits in ASD [30, 31]. We assessed whether MIA offspring have deficits in social interaction using three-chamber social tests. MIA offspring displayed significantly less social interaction behavior than the controls on both the social approach and social novelty tests at 4 w (Fig. 4a, b) and 8 w (Fig. 4e, f). Intriguingly, when prenatally pretreated with VAC, offspring spent more time with the mouse in the social approach test (Fig. 4a, e) and with the novel mouse in the social novelty test (Fig. 4b and f), suggesting that VAC pretreatment blocked the MIA-induced social impairment in the progeny. Meanwhile, unlike anxiety-related behavior, we showed that this preventive effect of VAC against social deficits induced by MIA continued beyond early development and into adulthood.

#### Depression-related behavior

Depression-related behavior was also identified in the FST and TST. Some patients with ASD appear to have elevated levels of depressive symptoms [32]. We found that in the FST, although not the TST, MIA offspring spent more time immobile than the controls did, at both 4 and 8 w (Fig. 4d, h; Fig. 4c, g). Overall, prior VAC treatment rescued depression-related behavior in the FST at 4 w (Fig. 4d) but not at 8 w (Fig. 4h).

### VAC mitigated LPS-associated aberrant brain cytoarchitecture and lamination in the embryonic neocortex

**Fig. 5:**
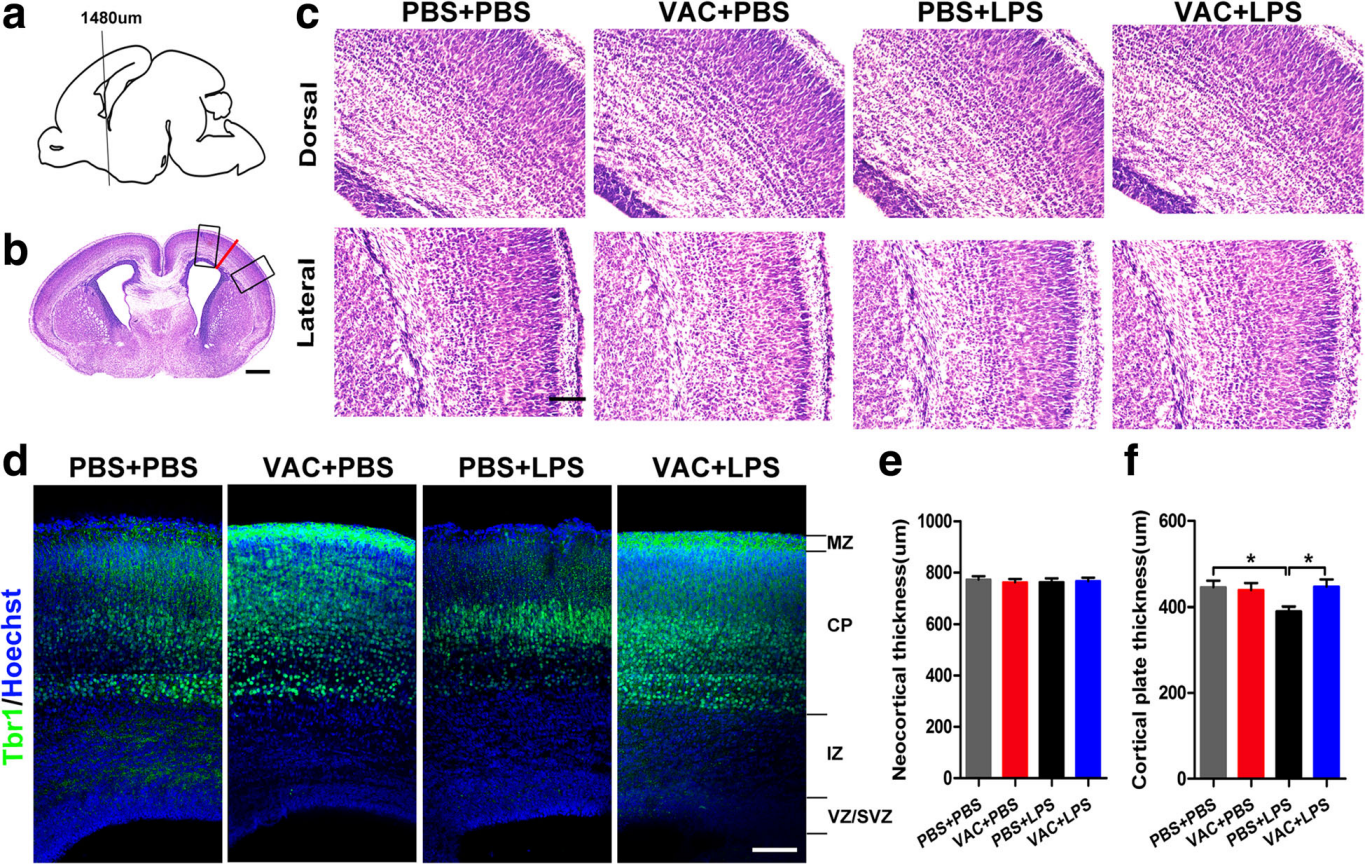
Maternal VAC and MIA effects on the thickness of the neocortex and cortical plate (CP) at E18.5. **a** Coronal sections of embryonic brains at 1480 μm from the front of the olfactory bulb were analyzed. **b** Coronal sections were Nissl stained. Dorsal and lateral regions are marked by the rectangles, and neocortical thickness is marked by the red line. **c** The dorsal and lateral views of the cortex are shown as the positions of the rectangles in **a**. **d** Coronal sections of the indicated embryonic brains at 1480 μm were immunostained with Tbr1 antibody (green) and Hoechst (nuclei). **e** There were no differences in neocortical thickness among the four groups. **f** A two-way ANOVA of CP thickness showed an interaction effect of MIA × VAC (*F*_1,20_ = 4.467, *p* = 0.047), and post hoc comparisons confirmed that MIA decreased the cortical plate thickness compared to that in controls (*p* = 0.016) and that VAC pretreatment restored the thickness to a normal level (*p* = 0.014). *n* = 6 mice/group; *p < 0.05 (two-way ANOVA and Bonferroni post hoc test). The results are all shown as the mean + s.e.m. Scale bars, 500 μm in **b**, 100 μm in **c**–**d**

Social behavior is regulated by specific brain areas, and abnormal fetal brain development has been reported in prenatally LPS-exposed mice [27]. Moreover, previous studies have drawn inconsistent conclusions regarding changes in embryonic cortical thickness and lamination in MIA models [17, 33]. To evaluate the effects of MIA on structural changes in the fetal brain, we used Nissl staining to analyze coronal sections of fetal brains. The locations of the dorsal and lateral regions in the cortex are shown in Fig. 5a, b. The data showed no significant differences in embryonic neocortical thickness between the MIA group and the controls (Fig. 5c, e).

Since the CP includes layers II–VI, and Tbr1 (T-box brain 1) is mainly expressed by post-mitotic neurons in layers V–VI [34], we used Tbr1 antibody staining in E18.5 cortex to more precisely define the thickness of the CP. Relative to controls, MIA offspring showed a significant decrease in CP thickness, while early application of VAC ameliorated this effect (Fig. 5d, f).

**Fig. 6:**
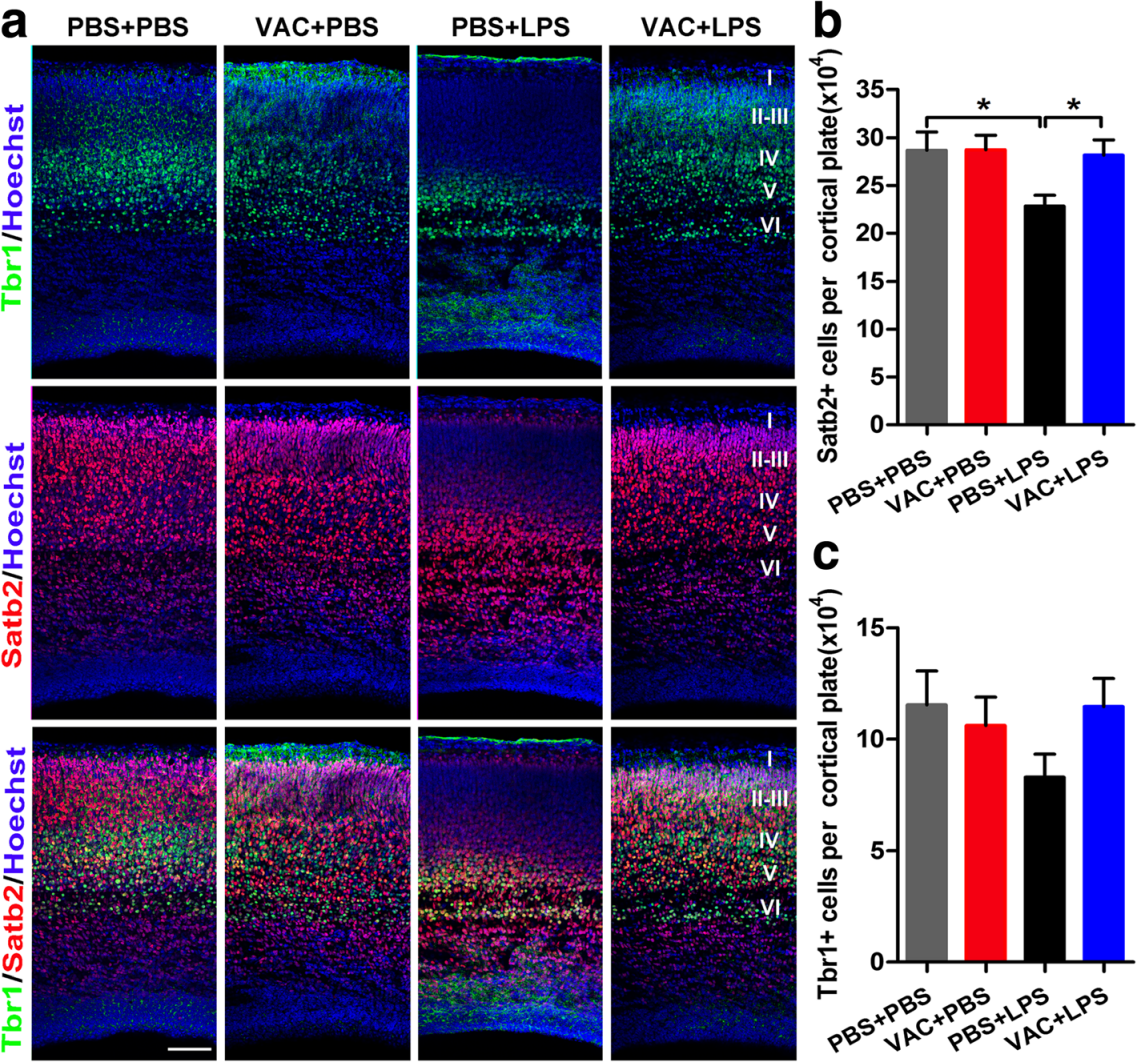
Maternal VAC and MIA effects on cortical lamination in the developing brain at E18.5. **a** Coronal sections of embryonic brains were immunostained with Tbr1 (green), Satb2 (red), and Hoechst (blue). Tbr1 mainly indicates the cells in cortical layers V–VI, while Satb2 labels layer II–V cells. Scale bar, 100 μm. **b** MIA induced a significant reduction of Satb2^+^ cells (*p* = 0.015), and VAC preprocessing reversed the effect (*p* = 0.025). **c** A similar but not significant trend was observed for Tbr1^+^ cells in the cortical plate (*p >* 0.05). *n* = 6 mice/group; *p < 0.05 (two-way ANOVA and Student’s *t* test). The results are all shown as the mean + s.e.m.

The mammalian brain contains a six-layer neocortex, and each layer has a specific function that is tightly controlled by gene expression during the embryonic period. Specific genes such as Tbr1 (T-box brain 1) and Satb2 (special AT-rich sequence binding protein 2) play important roles in different stages of neuronal differentiation, and disruptions in these genes lead to profound cortical malformation [35, 36]. Reports have shown that there are stereotypical alterations in cortical patterning in LPS-related MIA offspring [17, 33]. To examine the effects of VAC on LPS-associated embryonic neocortical deficits, we labeled different cortical layers in coronal fetal brain sections with antibodies targeting Tbr1 (layers V–VI) and Satb2 (layers II–V). The results showed a significant decrease in Satb2^+^ cells (Fig. 6a, b) and a noticeable but nonsignificant decrease in Tbr1^+^ cells (Fig. 6a, c; *p* = 0.09) in LPS-exposed neocortex, suggesting that LPS-induced neuronal loss occurs mainly in upper layers. However, VAC pretreatment completely rescued the decline in Satb2^+^ cell counts (Fig. 6a, b) and showed a strong but nonsignificant tendency to rescue the deficits in Tbr1^+^ cells (Fig. 6a, c; *p* = 0.097).

### VAC rescued LPS-induced decreases in the numbers of IPCs in the developing neocortex and of astrocytes in the adult neocortex

**Fig. 7:**
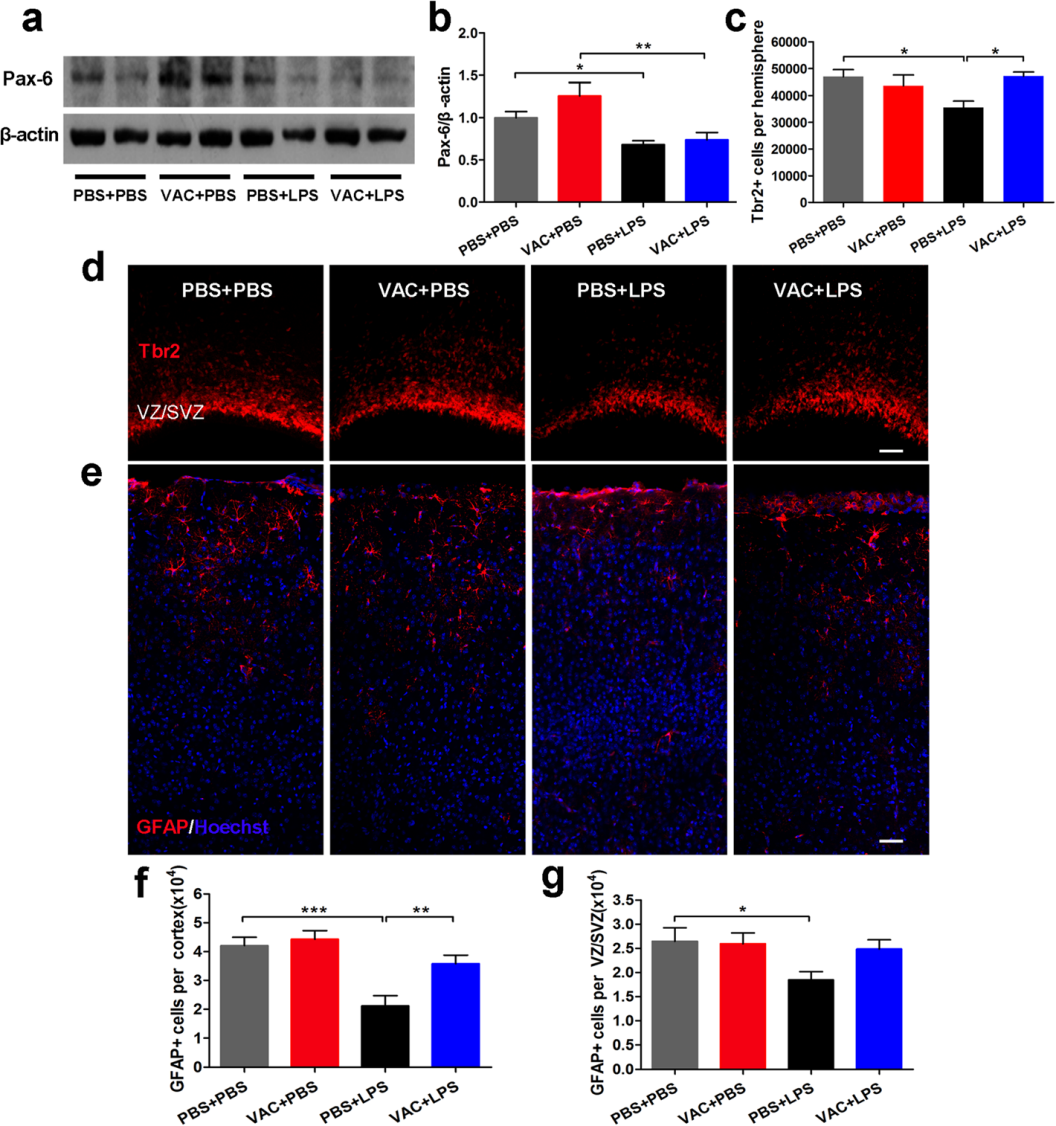
Maternal VAC and MIA effects on neural precursor cells in the embryonic cortex at E18.5 and astrocytes in the adult cortex at 6 weeks. **a** Representative Western blot bands of Pax-6 and β-actin. **b** The ratio of Pax-6 to β-actin was quantified. ANOVA showed a significant main effect of MIA (*F*_1,12_ = 17.899, *p* = 0.001) on Pax-6 concentration, and post hoc comparisons revealed that MIA reduced the Pax-6 concentration (*p* = 0.042) and that VAC pretreatment failed to rescue that effect (*p* > 0.05). **d** Coronal sections of E18.5 mouse brains were immunostained with Tbr2 (red), and VZ/SVZ images were obtained. **c** The number of Tbr2^+^ cells per hemisphere was counted. ANOVA showed an interaction effect between MIA and VAC (*F*_1,12_ = 7.252, *p* = 0.02), and post hoc comparisons confirmed that MIA decreased the number of Tbr2^+^ cells (*p* = 0.013) and that VAC pretreatment reversed the decrease (*p* = 0.012). **e** Coronal sections of 6-week-old mouse brains were immunostained for GFAP (red) and Hoechst (blue). Cortical images are shown. **f** There are significant main effects of MIA (*F*_1,12_ = 22.412, *p* = 0.0004) and VAC (*F*_1,12_ = 7.395, *p* = 0.019) for GFAP^+^ cells. Post hoc comparisons showed that MIA induced a significant reduction of astrocytes in the cortex (*p* < 0.001) and that VAC pretreatment prevented the effect (*p* = 0.006). **g** A trend toward a similar effect for the VZ/SVZ was also observed. *n* = 4 mice/group; *p < 0.05, **p < 0.01, ***p < 0.001 (two-way ANOVA and Bonferroni post hoc test). The results are all shown as the mean + s.e.m. Scale bars, 50 μm in (**d**, **e**)

The transcription factors Pax6, Tbr2, and Tbr1 are expressed sequentially by RGCs, IPCs, and postmitotic neurons in the developing neocortex [37]. In the present study, to determine whether abnormal neuronal differentiation contributes to dysfunctional lamination in the embryonic cortex, the protein extracts were separated from the fetal cortex at E18.5, and Pax-6 protein was detected. In addition, Tbr2^+^ cells were counted in the fetal neocortex at E18.5. The data revealed that the Pax-6 level and Tbr2^+^ cell number were significantly lower in the MIA offspring than in the controls (Fig. 7a–d); VAC pretreatment rescued only the number of Tbr2^+^ cells (Fig. 7c, d) and not the Pax-6 level in MIA offspring (Fig. 7a, b). In addition, cell apoptosis and proliferation were measured by quantifying cleaved caspase-3 protein (CC3) and PCNA, respectively (Additional file 2: Figure S1G). The results showed that there were very few CC3^+^ cells present in the developing neocortex (Additional file 2: Figure S1A–E). MIA increased the concentrations of CC3 and PCNA in fetal cortex at E18.5, which was consistent with previous reports in LPS-induced MIA models [7, 38]. However, we found that VAC exerted no obvious effects on the increases in apoptotic cells and proliferative cells in MIA fetal neocortex (Additional file 2: Figure S1H and I), implying that apoptosis and proliferation may not contribute to the effects of VAC in this study. These results suggested that MIA induced aberrant lamination of the neocortex, at least partly attributable to differential reduction in IPCs and RGCs. Interestingly, VAC mitigated the aberrant lamination of the embryonic neocortex in MIA offspring by restoring IPC differentiation.

To further explore the influences of maternal VAC and LPS treatment on adult neocortex, we immunostained coronal sections from adult offspring with antibodies against NeuN for neurons, GFAP for astrocytes, and BDNF for brain-derived neurotrophic factor. The data revealed that LPS decreased the number of astrocytes (Fig. 7e, f) and the expression of BDNF (Additional file 3: Figure S2C and G) in the adult neocortex, whereas VAC counteracted these effects. Similar results of astrocytes were also observed in the VZ/SVZ (Fig. 7g). Unexpectedly, the number of neurons was comparable among groups (Additional file 4: Figure S2B).

### Changes in expression of relevant genes in the fetal cortex after VAC and LPS treatment

**Fig. 8:**
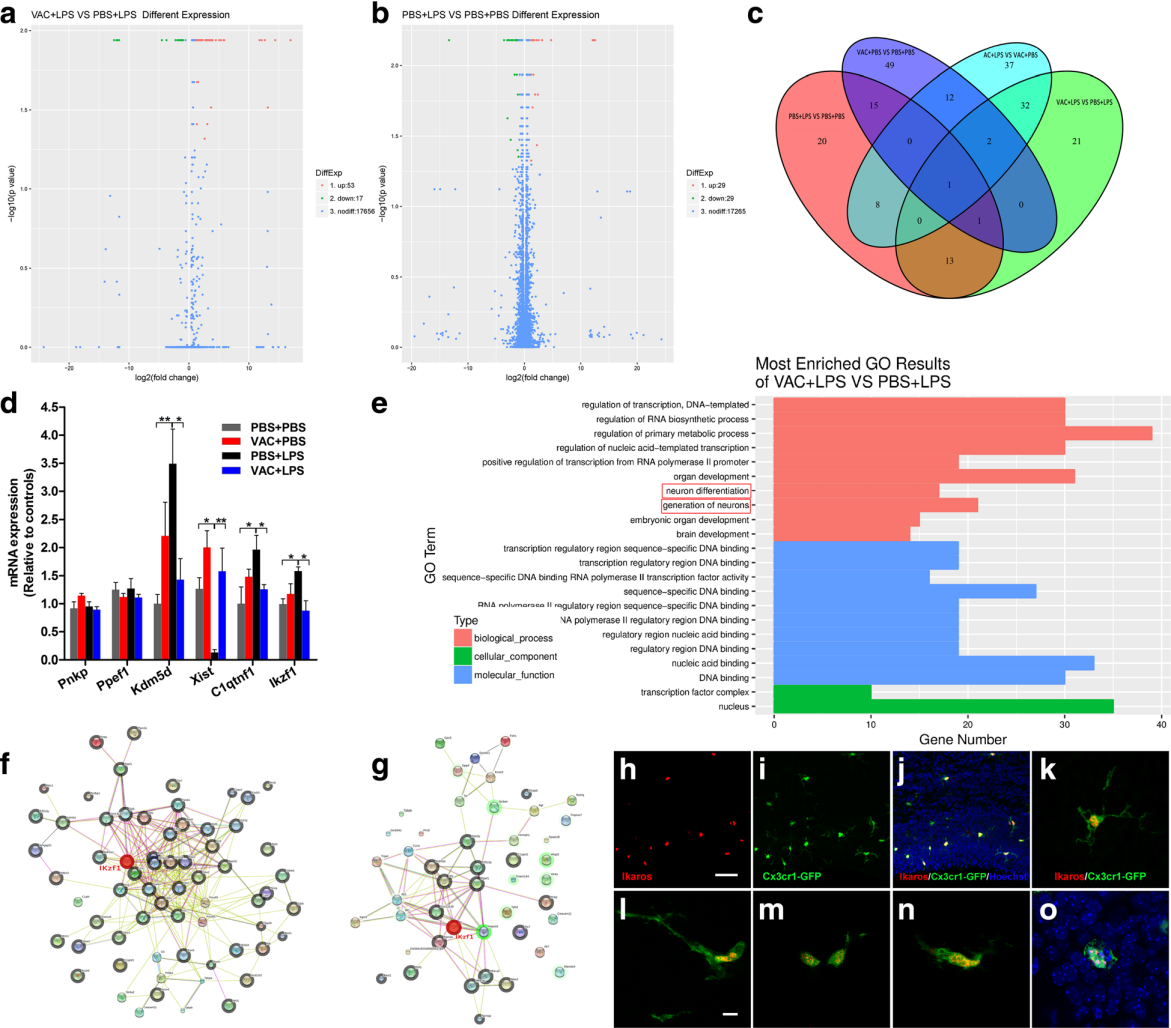
Whole-transcriptome analysis of maternal VAC and MIA effects on E18.5 cortical gene expression. **a** The volcano diagram includes 53 upregulated genes (red) and 17 downregulated genes (green) in VAC+LPS compared to PBS+LPS. **b** Volcano diagram includes 29 upregulated genes (red) and 29 downregulated genes (green) in PBS+LPS compared to PBS+PBS. **c** Venn diagram represents the overlap of differentially expressed genes among groups; 13 common differentially expressed genes were analyzed (*n* = 3 mice/group). **d** Real-time PCR analysis validated common differential gene expression among the four groups (*n* = 3 mice/group). There was an interaction effect between MIA and VAC (*F*_1,8_ = 10.075, *p* = 0.013) for Ikzf1, and post hoc comparisons showed that MIA increased Ikzf1 expression compared to controls (*p* = 0.018) and that VAC pretreatment rescued the Ikzf1 expression (*p* = 0.007). **e** Most-enriched GO terms for VAC+LPS vs PBS+LPS. The enriched biological processes included neuron differentiation, generation of neurons, and brain development. The protein interaction networks for VAC+LPS vs PBS+LPS (**f**) and VAC+LPS vs PBS+LPS (**g**) are shown. The red circles in **f** and **g** represent the protein Ikzf1 (Ikaros), which holds core positions in the networks. **h–j** Images of Ikaros (red) and Cx3Cr1-GFP^+^ (green) microglia in the VZ/SVZ of the fetal brain. **k–o** High-magnification images of the colocalization of Ikaros (red) and various morphological forms of microglia (green). **k** An early branched form. **l** A more branched form. **m–o** An amoeboid form. *p < 0.05, **p < 0.01 (two-way ANOVA and Bonferroni post hoc test). The results are all shown as the mean ± s.e.m. Scale bars, 50 μm in **h–j**, 10 μm in **k–o**

To gain mechanistic insight into the regulatory effect of VAC pretreatment on cortical lamination, we conducted an RNA-Seq assay to determine relevant gene expression in the cerebral cortex. The numbers of genes showing differential expression (q-value| FDR ≤ 0.05) between groups are presented in Fig. 8a, b. A Venn diagram was constructed to present the overlap of these differential genes among groups, and 13 genes were related to the effects of VAC treatment on the MIA fetal brain (Fig. 8c). Importantly, based on Gene Ontology (GO) enrichment results, we identified neuronal differentiation as one of the biological processes showing the greatest differences between VAC+LPS and PBS+LPS mice, which confirmed the effect of VAC on MIA offspring at the gene level and was in accordance with our histomorphology results (Fig. 8e).

Next, qRT-PCR was performed to analyze these candidate genes, and four differentially expressed genes were confirmed to be related to the ability of VAC to protect against social deficits and abnormal cortex development in MIA mice (Fig. 8d). Of the four genes, Ikzf1 (IKAROS family zinc-finger 1) was notable because it not only participated in the process of neuronal differentiation but also played a central role in the interaction network of proteins (Fig. 8f, g). Expression of Ikaros (Ikzf1), which promotes the differentiation of early-born cortical neurons, is high in mouse cortical progenitor cells at early stages and then decreases over time [39–41]. Notably, overexpression of Ikaros results in abnormal lamination during the late embryonic stages [41]. Ikaros copy number variants have reportedly been identified in ASD probands [42]. Consistently, our data showed that LPS mice had higher Ikzf1 expression than controls at a late stage (E18.5) and that VAC preconditioning rescued its expression (Fig. 8d; *p* < 0.05), supporting a vital role for Ikzf1 in the effect of VAC pretreatment on abnormal lamination and autism-like behaviors. Intriguingly, Ikaros was located in the neocortex and VZ/SVZ (Fig. 8h–j) and colocalized with various morphological forms of CX3Cr1-GFP^+^ microglia (Fig. 8k–o), suggesting that microglia in the cerebral cortex could express Ikaros. Although Ikaros is well described in the lymphoid system and neuronal precursors, our findings extend the known biological functions of Ikaros to regulating cortical lamination from neuronal precursors to microglia.

### Pretreatment with VAC ameliorated the microglial inflammatory response to LPS challenge

**Fig. 9:**
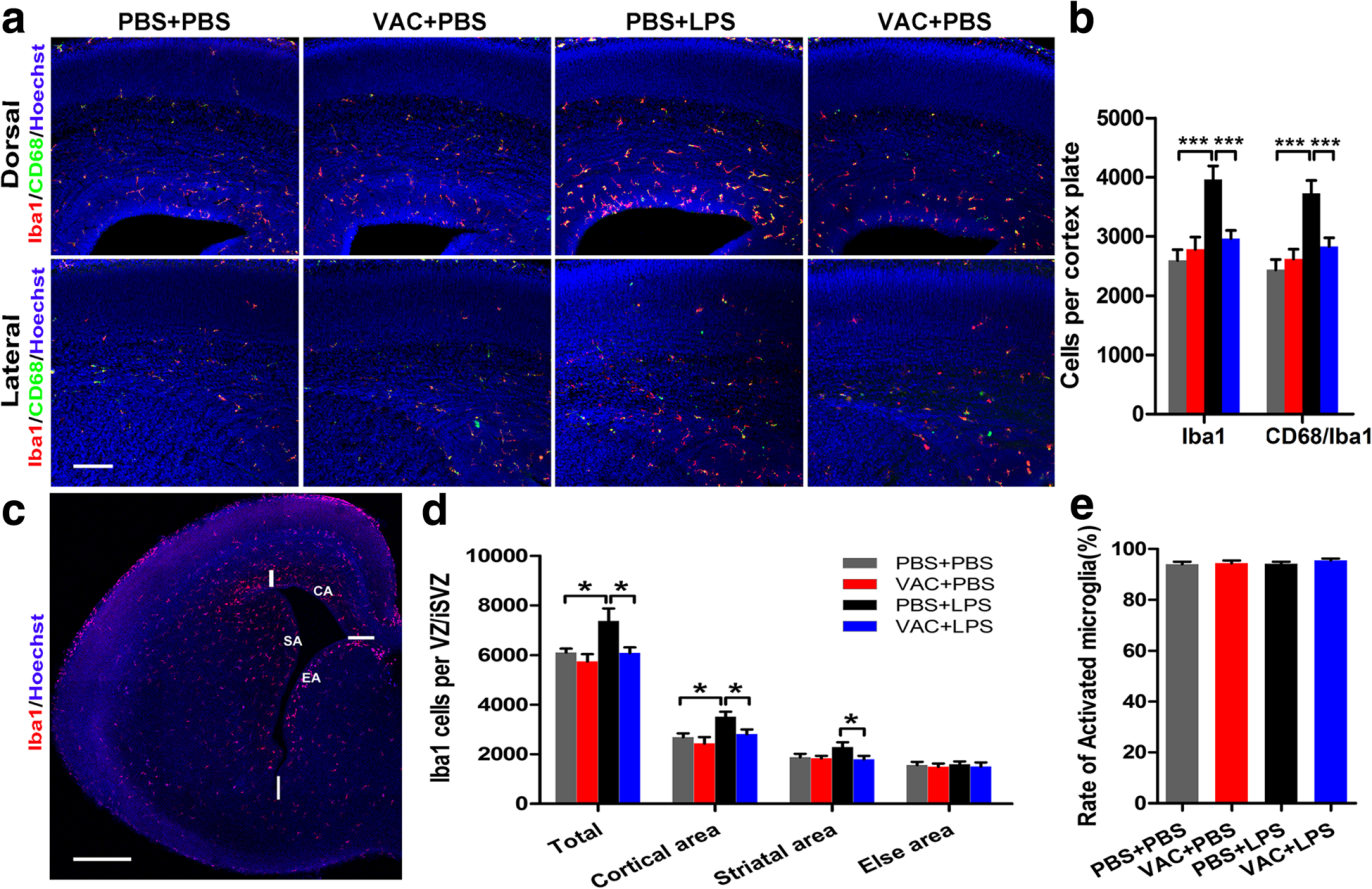
Effects of maternal VAC and MIA on central inflammatory response at E18.5. Representative images of staining with Iba1 (red), CD68 (green), and Hoechst (blue) in the cortical (**a**) and hemispherical (**c**) areas in fetal brains at E18.5. **b** ANOVA showed a significant main effect of MIA (*F*_1,12_ = 16.135, *p* = 0.002) and interaction effect between MIA and VAC (*F*_1,12_ = 9.479, *p* = 0.01) for the number of Iba1^+^ cell. Similar result was shown for CD68^+^ cells, main effect of MIA: *F*_1,12_ = 17.996, *p* = 0.001; interaction effect between MIA and VAC: *F*_1,12_ = 9.311, *p =* 0.01. Post hoc test revealed that VAC pretreatment rescued the MIA-induced accumulation of Iba1^+^ cells and CD68^+^ cells in the cortical plate (*p* < 0.001). **d** Similar results were observed in the VZ/SVZ, especially in cortex-related areas. There were significant main effects of MIA (*F*_1,12_ = 6.170, *p* = 0.029) and VAC (*F*_1,12_ = 6.332, *p* = 0.027) for total Iba1^+^ cells in VZ/SVZ. A significant main effect of LPS (*F*_1,12_ = 8.411, *p* = 0.013) and VAC (*F*_1,12_ = 5.219, *p* = 0.049) for Iba1+ cells was quantified in the CA. **e** The ratio of CD68^+^ cells to Iba1^+^ cells (the proportion of activated microglia) did not differ among the four groups (*p* > 0.05). *n* = 4 mice/group; *p < 0.05, ***p < 0.001 (two-way ANOVA and Bonferroni post hoc test). The results are all shown as the mean + s.e.m. Scale bars, 100 μm in **a**, 500 μm in **c**

Microglial priming has been proposed to be a major consequence of MIA and represents a critical link in a causal chain that leads to a wide spectrum of neuronal dysfunctions and behavioral phenotypes [43]. To determine whether VAC pretreatment could influence microglia in the developing brain after LPS challenge, we observed the Iba1^+^ cells (microglia) and Iba1^+^/CD68^+^ cells (activated microglia) located in the dorsal and lateral cortex of the fetal brain at E18.5 (Fig. 9a). Quantitative analysis with a stereology system showed more microglia (Iba1^+^ cells) and activated microglia (Iba1^+^/CD68^+^ cells) in MIA offspring than in controls, and preconditioning with VAC restored the numbers to normal levels (Fig. 9b). Numerous microglia were also observed to be located in the VZ/SVZ, which was divided into several subsets—cortical area (CA), striatal area (SA), and else areas (EA)—for quantification (Fig. 9c). Our data showed that VAC had a similar effect on microglia in the VZ/SVZ of MIA mice, especially in the CA (Fig. 9d), suggesting that VAC could rescue the LPS-induced inflammatory response in the brain. Additionally, the ratio of activated microglia (ratio of CD68/Iba1^+^ cells to Iba1^+^ cells) averaged 90% and did not differ among groups (Fig. 9e). Furthermore, the activation of microglia paralleled the change in Ikaros expression in the present study.

Given that microglia are reported to phagocytose neural precursor cells in the developing neocortex [44], we next detected microglia phagocytosing IPCs by colocalization of Tbr2 and CD68 in the fetal neocortex at E18.5 (Additional file 4: Figure S3E–J). Quantitative analysis revealed that LPS promoted microglial phagocytosis of IPCs, and this effect was mitigated by pretreatment with VAC (Additional file 4: Figure S3A–D and K), suggesting that VAC rescued the number of IPCs not only by restoring IPC differentiation but also by attenuating microglial phagocytosis. Nonetheless, the molecular mechanism of this phagocytosis has not yet been clarified, and our results provide evidence for the daring conjecture that Ikaros expression may be correlated with the microglial phagocytosis of neural precursor cells.

## Discussion

Herein, we show that VAC attenuated the deleterious effects induced by MIA, including its effects on fetal loss, social behaviors, thickness of the CP, and abnormal neocortical lamination. Importantly, these effects are closely associated with the fact that VAC promotes differentiation of neural progenitor cells, a phenomenon that may involve microglia-derived Ikaros. VAC rescued the abnormal Ikaros expression, microglial proliferation, and activation in MIA offspring. This work highlights the key preventive impact of VAC on LPS-caused autism-related impairments during brain development.

Social interaction behavior is associated with some specific brain areas, especially the cortex [45, 46]. Interestingly, our study showed that social deficits were related to decreased thickness of the fetal CP in LPS-induced MIA offspring, which was consistent with previous reports of reduced cortical volume [47] or thickness [33] in similar ASD models. However, there were conflicting results from other ASD models [17, 48], and this discrepancy may be due to differences in infection models and target animal species [7, 49]. Further, we found aberrant lamination of the embryonic neocortex and reduced neuron counts, especially in the upper layers, which contributed to the reduction in CP thickness. The development of the six-layer neocortex in rodents is known to start at E11.5 in an inside-out pattern [50]. Thus, the upper layers were more susceptible than deeper layers when maternal immunity was mobilized by LPS at E14.5. Overall, VAC attenuated the deleterious effects of MIA on thickness and lamination, which mainly manifested in the restored neuronal numbers in the upper layers. These effects on cortical layers might be associated with apoptosis or proliferation [7]; however, we confirmed that there was a negligible effect on apoptotic factors, reflected by a slight increase in CC3^+^ cells in MIA offspring, and VAC had no protective effect on apoptosis. Moreover, similar results for proliferation were obtained in the embryonic neocortex. Consequently, apoptosis and proliferation were excluded as causes of the recovery in neuron number for VAC.

Another possible explanation for the alteration of neuron number may be abnormal neuronal differentiation [51]. The numbers of RGCs and IPCs were decreased in the embryonic neocortex of MIA models, but VAC pretreatment rescued the loss of IPCs. These data thus confirmed our conjecture that VAC prevents the effects of LPS on the developing cortex, a phenomenon that was linked to the decreased autism-like behavior. However, our data are not consistent with the results observed in an autism-specific maternal autoantibody infection model [52], which may be because of autoantibody-induced increases in stem cell proliferation, whereas MIA might affect RGC differentiation and have little effect on proliferation. Moreover, our in-depth experiments revealed that the restorative effects of VAC on the number of IPCs in the MIA fetal cortex were due to the increase in IPC differentiation and the decrease in microglial phagocytosis of IPCs. Later, in adult MIA offspring (6 weeks), neither VAC nor LPS influenced the numbers of neurons, but VAC increased the BDNF expression in the MIA cortex through other types of cells, such as astrocytes, to provide more trophic support to neurons. Therefore, neuronal functions in adulthood, such as neuronal plasticity, remain to be explored in future research. In addition, the decreased number of astrocytes in the adult brain differed from the reports that two injections of LPS (500 μg/kg) led to an increased number of astrocytes in 8-day-old rat pups [53]. This inconsistency may be because the present study used a milder stimulus (LPS, 75 μg/kg) than the previous study, meaning that the dose of LPS used in this study might induce treated animals to produce fewer RGCs than controls.

The development of a six-layer neocortex is coordinated by specialized genes programming neurogenesis and neuronal differentiation, which was confirmed by the pathways identified in our study as most enriched according to GO analysis. Ikzf1 was the most important gene showing differential expression among the four groups. Reports have shown that Ikzf1 (Ikaros) is expressed in cortical progenitor cells and helps determine the fates of early-born neurons in the cerebral cortex [41]. Therefore, Ikzf1 is a promising candidate gene to mediate the effect of VAC treatment on MIA models. In addition, Ikzf1/Ikaros seems to have a complex role in neuronal differentiation. Ikaros was previously reported to be a tumor suppressor and pro-differentiation factor [54, 55]. By contrast, a recent study revealed that Ikaros overexpression decreased the number of upper-layer neurons in the cortex at E17.5 [41]. Consistent with this result, we found that MIA induced an increase in the expression of Ikzf1 at E18.5, accompanied by a decrease in neurons in the upper layers, and VAC pretreatment alleviated these effects. Unexpectedly, the total number of neurons showed no difference across the four groups in adulthood. One explanation may be that LPS-induced sustained Ikzf1 expression results in a delayed appearance of upper-layer neurons and that the disorganized laminar structure returns to normal after birth. Alteration in the migration of newborn cells has previously been linked to autism [52]. Another explanation may be that overexpressed Ikzf1 extends the window of deeper-layer neurogenesis [41], so that the overall number of neurons in the cortex in MIA mice became normal during adulthood. In addition, Ikaros/Ikzf1 gene-deficient mice displayed behavioral alterations in an antidepressive phenotype [56], implying that Ikaros is relevant to depression-related behavior, which is consistent with our results from the FST and TST. Taken together, our data provide evidence for the crucial role of Ikzf1 in determining cortical lamination and the development of autism-like behaviors in the late stage of prenatal development. Importantly, VAC pretreatment prevented MIA-induced social deficits and aberrant lamination by promoting neuronal differentiation, which depended on restoring normal Ikaros expression. Moreover, we found that Ikzf1/Ikaros was expressed in microglia, and microglial proliferation and activation paralleled the expression of Ikaros. Although an immune-mediator signaling cascade was reported to upregulate the Ikzf1 gene specifically by activating the upstream protein STAT3 in BV-2 cells [57], the specific mechanism whereby Ikaros regulates the differentiation of cortical progenitor cells still needs further investigation.

VAC prevents the effects of LPS challenge on neuronal progenitor cells, microglial activation, and the numbers of astrocytes. Therefore, we hypothesize that VAC exerted protective effects through the following mechanisms. VAC-induced M2 microglial/macrophage polarization developed a pro-neurogenic niche via astrocytic secretion of BDNF (Fig. 7e and Additional file 3: Figure S2G) and microglial expression of IGF-1 [13, 15]. Moreover, a recent report revealed that Ikaros is expressed in progenitor cells and regulates early-born neuronal fates [41]. Ikaros overexpression in neuronal progenitor cells induced developmental defects, including disrupted cortical lamination. In line with our results, LPS-treated fetal brain showed sustained Ikaros expression, which could be inhibited by VAC. However, our findings show that microglia, not neurons, could express Ikaros in the cerebral cortex, reflected by colocalization with CX3Cr1-GFP^+^ microglia (Fig. 5k). Because the Ikaros family of transcription factors has critical functions in immune regulation [58], VAC may regulate microglial inflammation via Ikzf1. Thus, we conclude that VAC indirectly influences cortical lamination after maternal immune activation, through a mechanism involving Ikaros.

It has been reported that the cytokine IL-17a, which is relevant to microglial activation and proliferation [59], promotes abnormal cortical development and ASD-like behavioral phenotypes [60]. However, we failed to detect any alteration in IL-17a in the cortex of E18.5 mice after VAC or MIA. One possible reason is that cytokines may have been transiently altered after maternal LPS exposure [53, 61] and that IL-17a expression was restored to normal levels by E18.5. Another possible reason is that, unlike in the polyinosinic-polycytidylic acid-induced autism model [60], IL-17a may be not sufficient to cause aberrant lamination and social deficits in this LPS-induced MIA model. Therefore, more information about cytokines will be necessary in the future.

## Conclusions

Overall, for the first time, we show that VAC pretreatment in early gestation is sufficient to prevent impairments in social interaction and lamination in an MIA-induced autism model and that this process is associated with neuronal differentiation and Ikzf1 gene. Our findings strongly suggest that influenza vaccination in early pregnancy is a potential preventive measure against autism induced by maternal bacterial infection.

## Additional files

Additional file 1: RNA-seq procedures. (DOCX 22 kb)

Additional file 2: **Figure S1.** Effects of maternal VAC and MIA on cell apoptosis and proliferation in the developing neocortex at E18.5. (A) Low-magnification image of CC3^+^ cells (red). (B-E) High-magnification images of CC3^+^ cells (red). All nuclei are labeled with Hoechst (blue). Scale bar, 50 μm. (F) Low-magnification image of PCNA^+^ cells (red) showed that a majority of PCNA^+^ cells were located in the VZ/SVZ. (G) A representative blot of CC3 and PCNA is shown. Equal loading of proteins is illustrated by the β-actin bands. (H) The group densitometry analysis of CC3 protein. There was a main effect of MIA (*F*_1,12_ = 26.065, *p* < 0.001) and post hoc test showed increases in MIA (*p* = 0.003) and VAC+LPS (*p* = 0.004) mice compared with controls. (I) The group densitometry analysis of PCNA. There was a main effect of MIA (*F*_1,12_ = 14.42, *p* = 0.003) and post hoc test showed increases in the MIA group (*p* = 0.003), but VAC pretreatment had a trend toward preventing this effect (*p* = 0.288). *n* = 4 mice/group; **p < 0.01 (two-way ANOVA and Bonferroni post hoc test). The results are all shown as the mean + s.e.m. Scale bar, 50 μm in A-E, 500 μm in F. (TIF 2162 kb)

Additional file 3: **Figure S2.** Effects of maternal VAC and MIA on neurons and BDNF in the cerebral cortex of animals at 6 weeks. Coronal sections of indicated 6 week mouse brains were immunostained for NeuN and BDNF. (A–B) Stereological analysis of NeuN^+^ cells (red) in the neocortex revealed that the number of NeuN^+^ cells (neurons) did not differ among the four groups. (C) The position of the NeuN (red) and BDNF images (green) in the adult cortex is shown as the position of the semitransparent white frame in (A). (D–F) High-magnification images of the colocalization of BDNF and neurons. (G) Quantifications of the integrated density (IntDen) of BDNF. ANOVA showed a significant main effect of LPS (*F*_1,8_ = 40.109, *p* = 0.0002) and VAC (*F*_1,8_ = 7.410, *p* = 0.026) for BDNF. Post hoc analysis showed a decreased abundance of the protein in MIA offspring (*p* = 0.001), but VAC preprocessing rescued this effect (*p* = 0.019). *n* = 4 mice/group; *p < 0.05, **p < 0.01 (two-way ANOVA and Bonferroni post hoc test). The results are all shown as the mean + s.e.m. Scale bar, 100 μm in A and C, 10 μm in D–F. (TIF 2085 kb)

Additional file 4: **Figure S3.** Effects of maternal VAC and MIA on phagocytosis of neural precursor cells by microglia in the developing neocortex at E18.5. (A–D) Representative images of staining for TBR2 (red) and CD68 (green) in the VZ/SVZ of fetal mouse brains from the four groups. (E) Staining with Tbr2 (red), CD68 (green), and Hoechst (blue) in the cortical and VZ/SVZ areas. Scale bar, 100 μm. (F–J) CD68^+^ microglia (green) in the SVZ contact and envelope Tbr2^+^ neural precursor cells (red). Scale bar, 20 μm. (K) Quantifications showed an increase in the number of neural precursor cells being targeted by microglia (*p* = 0.042), and VAC pretreatment rescued the effect (*p* = 0.024). *n* = 4 mice/group; * *p* < 0.05, ** *p* < 0.01 (two-way ANOVA and Student’s *t* test). The results are all shown as the mean + s.e.m. Scale bar, 100 μm in A–E, 20 μm in F–J. (TIF 2478 kb)

## Abbreviations

BDNF: Brain-derived neurotrophic factor
BSA: Bovine serum albumin
CP: Cortical plate
IL-17a: Interleukin-17a
IPCs: Intermediate progenitor cells
IZ: Intermediate zone
LPS: Lipopolysaccharide
MIA: Maternal immune activation
OFT: Open field test
PBS: Phosphate-buffered saline
PMSF: Phenylmethylsulfonyl fluoride
RGCs: Radial glial cells
RIPA: Radio-immunoprecipitation assay
SVZ: Subventricular zone
VAC: Influenza vaccination
VZ: Ventricular zone
