# Supplementary material for: Prenatal influenza vaccination rescues impairments of social behavior and lamination in a mouse model of autism
Source: J Neuroinflammation. 2018 Aug 13;15:228. doi: 10.1186/s12974-018-1252-z (PMC6090662; doi:10.1186/s12974-018-1252-z)
Supplement: Supplementary file 1 — RNA-seq procedures. (DOCX 22 kb) [file 12974_2018_1252_MOESM1_ESM.docx]

**Table S1** List of primer sequences used for QRT-PCR.

| **GENE** | **SEQUENCE** | |
| --- | --- | --- |
|  | **FORWARD** | **REVERSE** |
| **Gapdh** | AGGAGAGTGTTTCCTCGTCCC | TGCCGTTGAATTTGCCGTGA |
| **IL-17a** | CTCCAGAAGGCCCTCAGACTAC | AGCTTTCCCTCCGCATTGACACAG |
| **Pnkp** | TTCGTGGGAGATGCAGCAGG | TCGGGTCGAAAGCTGGAAGC |
| **Ppef1** | AGAAGGCAGCAACCGAGGAG | AATGCCCACTCGGCCAATGA |
| **Kdm5d** | ATTCCGCGATCCTCTTGGCT | TGGGCCTCCAGTTCATTTAGC |
| **Xist** | TAAGGACTACTTAACGGGCT | TACTCAGACATTCCCTGGCA |
| **C1qtnf1** | GCCGGAAGAAGGCTTTGCAC | CCAAGTGTGCACGTTGAGGC |
| **Ikaros** | CCTCTGGAGCACAGCAGAACT | TCCTGACAAAGCCGAGCTGC |

**Supplemental method**

mRNA-seq

1. Library Preparation Process

1.1.RNA quality examination

1. RNA purity was detected by NanoDrop^®^ spectrophotometers (Thermo Fihser, MA, USA).
2. RNA concentration was measured using Qubit^®^ RNA Assay Kit in Qubit^®^ 3.0 Flurometer (Life Technologies, CA, USA).
3. RNA integrity was assessed using the RNA Nano 6000 Assay Kit of the Bioanalyzer 2100 system (Agilent Technologies, CA, USA).

1.2.Library preparation for mRNA-seq

A total amount of 1μg qualified RNA per sample was used as input material for the library preparation. The sequencing libraries were generated using the VAHTS mRNA-seq v2 Library Prep Kit for Illumina^®^ (Vazyme, NR601) following manufacturer’s recommendations. Firstly, mRNA was purified from total RNA using poly-T oligo-attached magnetic beads. Fragmentation was performed using divalent cations under elevated temperature in Vazyme Frag/Prime Buffer. The cleaved RNA fragments were copied into first strand cDNA using reverse transcriptase and random primers. Second strand cDNA synthesis was subsequently performed using buffer, dNTPs, DNA polymerase I and RNase H. Then, the cDNA fragments were end repaired with the addition of a single ‘A’ base at the 3'-end of each strand, ligated with the special sequencing adapters (Vazyme, N803) subsequently. The products were purified and size selected with VAHTS^TM^ DNA Clean Beads (Vazyme, N411) in order to get appropriate size for sequencing. PCR was performed and aimed products were purified finally.

Library examination

Library concentration was measured using Qubit^®^ RNA Assay Kit in Qubit^®^ 3.0 to preliminary quantify. Insert size was assessed using the Agilent Bioanalyzer 2100 system, and after the insert size consistent with expectations, qualified insert size was accurate quantified using qPCR by Step One Plus Real-Time PCR system (ABI, USA).

1.3.Library clustering and sequencing

The clustering of the index-coded samples was performed on a cBot Cluster Generation System (Illumia, USA) according to the manufacturer’s instructions. After cluster generation, the library preparations were sequenced on an Illumina Hiseq X Ten platform and 150bp paired-end module.

2.Analysis method

2.1.Quality control

The raw reads were filtered by removing reads containing adapter, ploy-N and low quality reads for subsequent analysis.

The steps of sequencing data filtering:

1) removing reads containing adapter;

2) removing reads containing ploy-N (i.e., unrecognized bases), reads with a ratio greater than 5%;

3) removing low-quality reads (the number of base which is Q≤10 is more than 50% of the entire read);

All the downstream analyses were based on clean data with high quality.

2.2.Mapping to the reference genome

The reference genome and gene model annotation files were downloaded directly from the genome website. The reference genome index was built using Bowtie2 (v2.2.9)[1], and paired-end clean reads were aligned to the reference genome using TopHat (v2.1.1)[2].

2.3.Transcriptome assembly

The mapped reads of each sample were assembled using Cufflinks (v2.2.1)[3] with a reference-based approach. This method employed spliced reads to determine exon connectivity. Cufflinks uses a probabilistic model to simultaneously assemble and quantify the expression levels of a minimal set of isoforms, which provides a maximum likelihood explanation of the expression data in a given locus.

2.4Quantification of gene expression levels

Cuffdiff (v1.3.0) was used to calculate FPKMs for coding genes in each sample. Gene FPKMs were computed by summing the FPKMs of the transcripts in each gene group. FPKM stands for “fragments per kilobase of exon per million fragments mapped”, and it is calculated based on the length of the fragments and the reads count mapped to each fragment.

2.5.Differential expression analysis

Cuffdiff (v2.2.1)[3] provides statistical routines for determining differential expression in digital transcript or gene expression datasets using a model based on a negative binomial distribution. Genes with corrected p values less than 0.05 and the absolute value of log2 (fold change) < 1 were assigned as significantly differentially expressed.

2.6.Gene Ontology (GO) and KEGG enrichment analysis

GO enrichment analysis of differentially expressed genes was implemented with perl module (GO::TermFinder)[4]. GO terms with corrected p value less than 0.05 were considered to be significantly enriched among the differentially expressed genes.

R functions (phyper and qvalue) were used to test for the statistical enrichment of the differentially expressed genes among the KEGG pathways. KEGG pathways with corrected pvalue less than 0.05 were considered to be significantly enriched among the differentially expressed genes.

2.7.Protein interaction network analysis

Protein interaction network analysis of differentially expressed genes were implemented based on StringDB database[5] if necessary.

Reference

[1] Langmead B, Salzberg S. Fast gapped-read alignment with Bowtie 2. Nature Methods. 2012, 9:357-359. [Bowtie 2]

[2] Kim D, Pertea G, Trapnell C, Pimentel H, Kelley R, Salzberg SL. TopHat2: accurate alignment of transcriptomes in the presence of insertions, deletions and gene fusions. .Genome Biology 2013, 14:R36. [TopHat2]

[3] Cole Trapnell, et al. Differential gene and transcript expression analysis of RNA-seq experiments with TopHat and Cufflinks. Nature Protocols 7, 562–578 (2012). [Cufflinks]

[4] Boyle EI, Weng S, Gollub J, Jin H, Botstein D, Cherry JM, Sherlock G. GO::TermFinder--open source software for accessing Gene Ontology information and finding significantly enriched Gene Ontology terms associated with a list of genes. Bioinformatics. 2004 Dec 12;20(18):3710-5. [GO::TermFinder]

[5] http://string-db.org/ [StringDB]
