# Supplementary figures and images for: Prenatal influenza vaccination rescues impairments of social behavior and lamination in a mouse model of autism
Source: J Neuroinflammation. 2018 Aug 13;15:228. doi: 10.1186/s12974-018-1252-z (PMC6090662; doi:10.1186/s12974-018-1252-z)

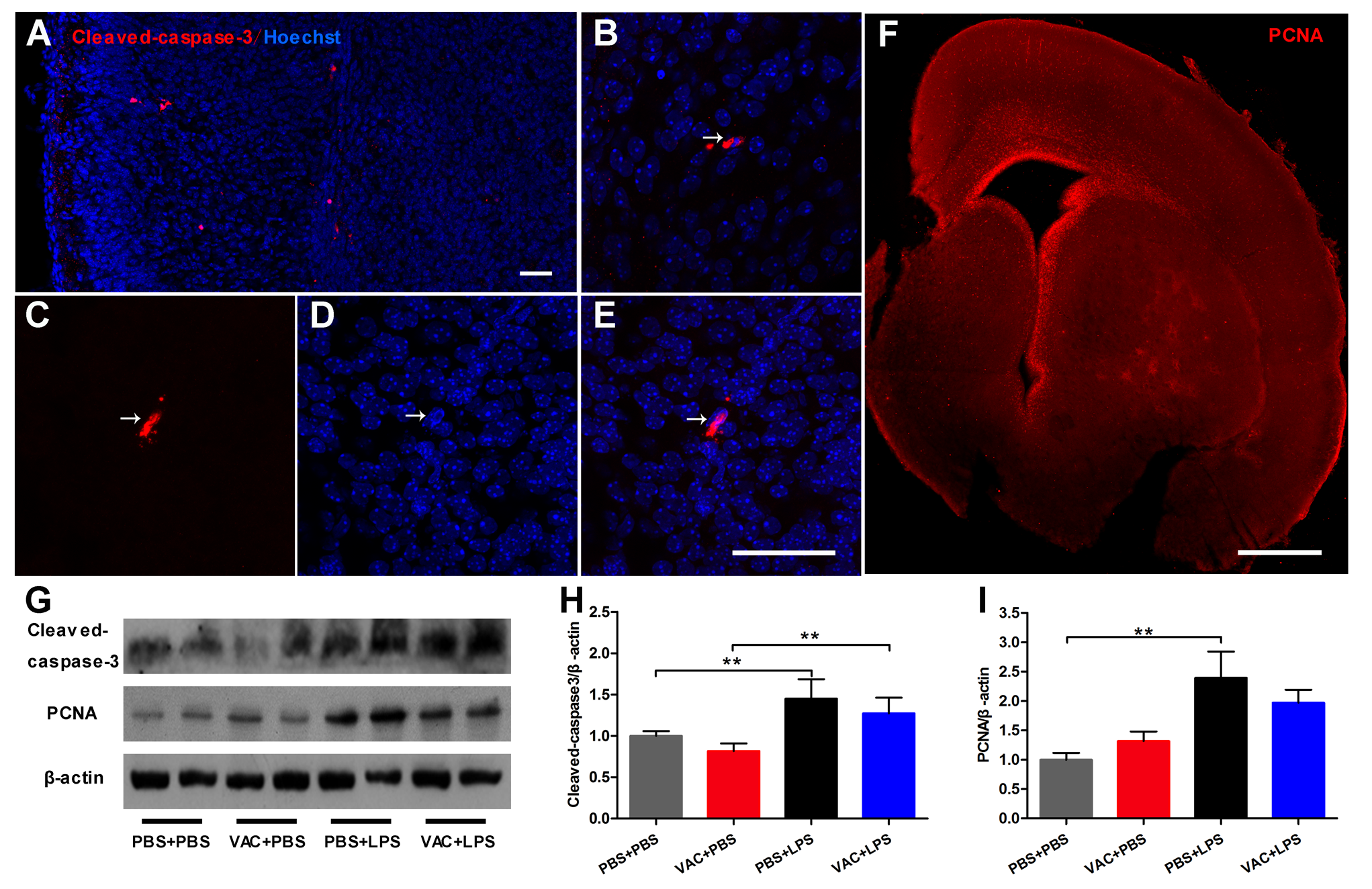

Supplement: Supplementary file 2 — Figure S1. Effects of maternal VAC and MIA on cell apoptosis and proliferation in the developing neocortex at E18.5. (A) Low-magnification image of CC3+ cells (red). (B-E) High-magnification images of CC3+ cells (red). All nuclei are labeled with Hoechst (blue). Scale bar, 50 μm. (F) Low-magnification image of PCNA+ cells (red) showed that a majority of PCNA+ cells were located in the VZ/SVZ. (G) A representative blot of CC3 and PCNA is shown. Equal loading of proteins is illustrated by the β-actin bands. (H) The group densitometry analysis of CC3 protein. There was a main effect of MIA (F1,12 = 26.065, p < 0.001) and post hoc test showed increases in MIA (p = 0.003) and VAC+LPS (p = 0.004) mice compared with controls. (I) The group densitometry analysis of PCNA. There was a main effect of MIA (F1,12 = 14.42, p = 0.003) and post hoc test showed increases in the MIA group (p = 0.003), but VAC pretreatment had a trend toward preventing this effect (p = 0.288). n = 4 mice/group; **p < 0.01 (two-way ANOVA and Bonferroni post hoc test). The results are all shown as the mean + s.e.m. Scale bar, 50 μm in A-E, 500 μm in F. (TIF 2162 kb) [file 12974_2018_1252_MOESM2_ESM.tif]

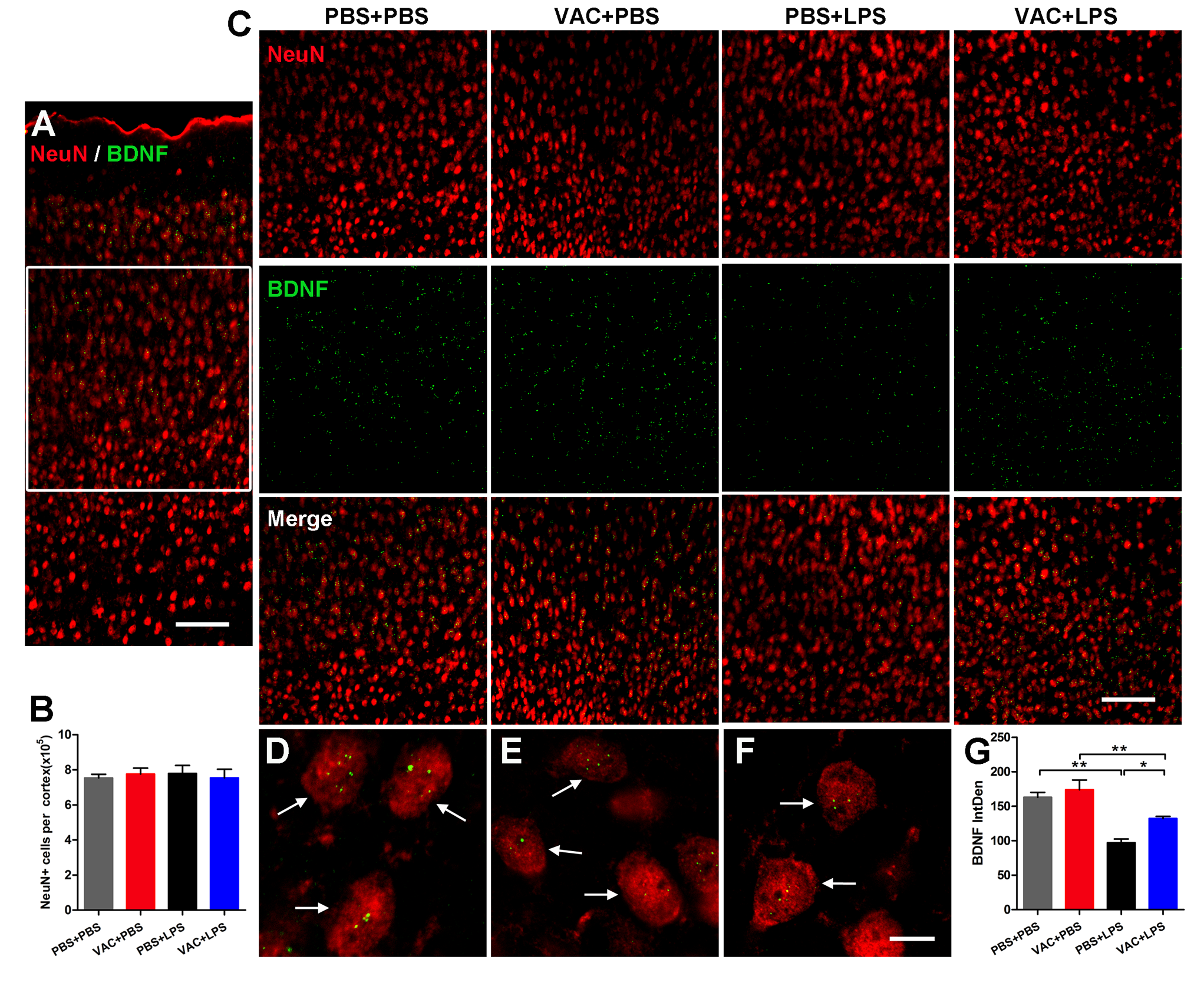

Supplement: Supplementary file 3 — Figure S2. Effects of maternal VAC and MIA on neurons and BDNF in the cerebral cortex of animals at 6 weeks. Coronal sections of indicated 6 week mouse brains were immunostained for NeuN and BDNF. (A–B) Stereological analysis of NeuN+ cells (red) in the neocortex revealed that the number of NeuN+ cells (neurons) did not differ among the four groups. (C) The position of the NeuN (red) and BDNF images (green) in the adult cortex is shown as the position of the semitransparent white frame in (A). (D–F) High-magnification images of the colocalization of BDNF and neurons. (G) Quantifications of the integrated density (IntDen) of BDNF. ANOVA showed a significant main effect of LPS (F1,8 = 40.109, p = 0.0002) and VAC (F1,8 = 7.410, p = 0.026) for BDNF. Post hoc analysis showed a decreased abundance of the protein in MIA offspring (p = 0.001), but VAC preprocessing rescued this effect (p = 0.019). n = 4 mice/group; *p < 0.05, **p < 0.01 (two-way ANOVA and Bonferroni post hoc test). The results are all shown as the mean + s.e.m. Scale bar, 100 μm in A and C, 10 μm in D–F. (TIF 2085 kb) [file 12974_2018_1252_MOESM3_ESM.tif]

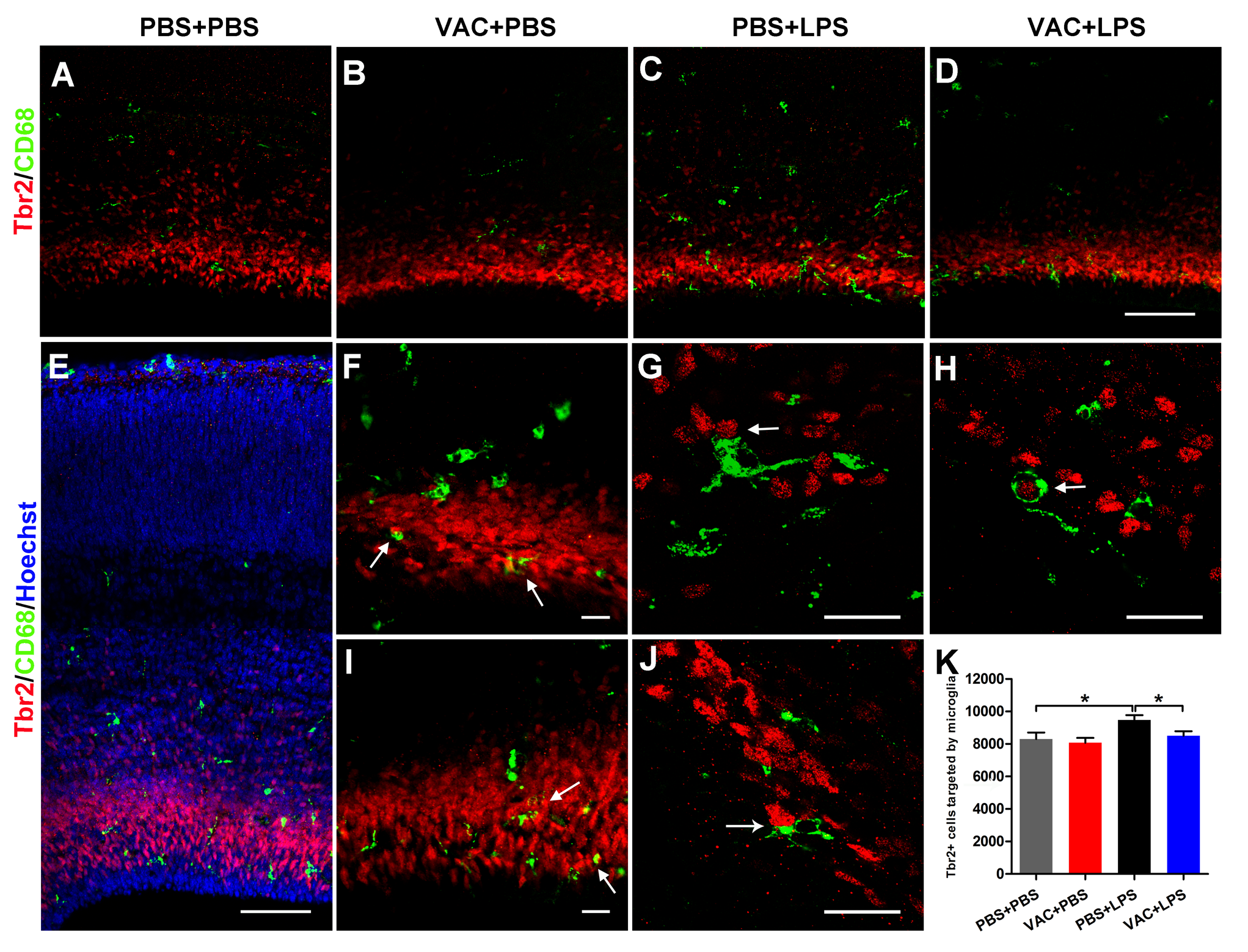

Supplement: Supplementary file 4 — Figure S3. Effects of maternal VAC and MIA on phagocytosis of neural precursor cells by microglia in the developing neocortex at E18.5. (A–D) Representative images of staining for TBR2 (red) and CD68 (green) in the VZ/SVZ of fetal mouse brains from the four groups. (E) Staining with Tbr2 (red), CD68 (green), and Hoechst (blue) in the cortical and VZ/SVZ areas. Scale bar, 100 μm. (F–J) CD68+ microglia (green) in the SVZ contact and envelope Tbr2+ neural precursor cells (red). Scale bar, 20 μm. (K) Quantifications showed an increase in the number of neural precursor cells being targeted by microglia (p = 0.042), and VAC pretreatment rescued the effect (p = 0.024). n = 4 mice/group; * p < 0.05, ** p < 0.01 (two-way ANOVA and Student’s t test). The results are all shown as the mean + s.e.m. Scale bar, 100 μm in A–E, 20 μm in F–J. (TIF 2478 kb) [file 12974_2018_1252_MOESM4_ESM.tif]
